# Dual-atom Pt heterogeneous catalyst with excellent catalytic performances for the selective hydrogenation and epoxidation

**DOI:** 10.1038/s41467-021-23517-x

**Published:** 2021-05-26

**Authors:** Shubo Tian, Bingxue Wang, Wanbing Gong, Zizhan He, Qi Xu, Wenxing Chen, Qinghua Zhang, Youqi Zhu, Jiarui Yang, Qiang Fu, Chun Chen, Yuxiang Bu, Lin Gu, Xiaoming Sun, Huijun Zhao, Dingsheng Wang, Yadong Li

**Affiliations:** 1 State Key Laboratory of Chemical Resource Engineering, Beijing Advanced Innovation Centre for Soft Matter Science and Engineering, Beijing University of Chemical Technology, Beijing, China; 2 Department of Chemistry, Tsinghua University, Beijing, China; 3 School of Chemistry and Chemical Engineering, Shandong University, Jinan, China; 4 Key Laboratory of Materials Physics, Centre for Environmental and Energy Nanomaterials, Anhui Key Laboratory of Nanomaterials and Nanotechnology, Institute of Solid State Physics, Chinese Academy of Sciences, Hefei, China; 5 Beijing Key Laboratory of Construction Tailorable Advanced Functional Materials and Green Applications, School of Materials Science and Engineering, Beijing Institute of Technology, Beijing, China; 6 Institute of Physics, Chinese Academy of Sciences, Beijing, China

**Keywords:** Heterogeneous catalysis, Materials science

## Abstract

Atomically monodispersed heterogeneous catalysts with uniform active sites and high atom utilization efficiency are ideal heterogeneous catalytic materials. Designing such type of catalysts, however, remains a formidable challenge. Herein, using a wet-chemical method, we successfully achieved a mesoporous graphitic carbon nitride (mpg-C_3_N_4_) supported dual-atom Pt_2_ catalyst, which exhibited excellent catalytic performance for the highly selective hydrogenation of nitrobenzene to aniline. The conversion of ˃99% is significantly superior to the corresponding values of mpg-C_3_N_4_-supported single Pt atoms and ultra-small Pt nanoparticles (~2 nm). First-principles calculations revealed that the excellent and unique catalytic performance of the Pt_2_ species originates from the facile H_2_ dissociation induced by the diatomic characteristics of Pt and the easy desorption of the aniline product. The produced Pt_2_/mpg-C_3_N_4_ samples are versatile and can be applied in catalyzing other important reactions, such as the selective hydrogenation of benzaldehyde and the epoxidation of styrene.

## Abstract (web summary)

Designing atomically monodispersed heterogeneous catalysts with uniform active sites and high atom utilization efficiency is of fundamental and practical interest. Here, the authors report a Pt2/mpg-C3N4 catalyst showing enhanced catalytic performance toward the selective hydrogenation and epoxidation

## Introduction

Atomically monodispersed heterogeneous catalysts with uniform active sites can be used as ideal models for understanding the correlations between compositions/structures and the corresponding performances, which are not only significant but also challenging in heterogeneous catalysis research^1–4^. Besides, atomically monodispersed catalysts also have high atom utilization efficiency and usually exhibit excellent catalytic activity^5–9^. For example, the well-known single-atom catalysts have been widely employed in many heterogeneous reactions^10–25^. Compared with the single-atom catalysts, dual-atom catalysts not only possess the same advantages of uniformity in the active sites and high atom utilization efficiency^26–29^, the involved two metal atoms can also cooperate and play a synergistic role in optimizing interactions between the active sites and the reactants or intermediates^30–35^. This may help to break the intrinsic linear scaling relationships between adsorption energies of reaction intermediates and further improve the catalytic performances. Although dual-atom heterogeneous catalysts possess so many unique advantages, synthesizing such materials remains a great challenge, which mainly comes from the difficulty in controlling the configuration uniformity of the active sites at the atomic scale.

Selective hydrogenation and epoxidation are two significant approaches to produce fine chemicals and high-value products in practical industrial applications^36,37^. These reactions include, for instance, the selective hydrogenation of nitroarenes to amines^38,39^, the selective hydrogenation of aldehyde compounds to alcohol compounds^40,41^, and the epoxidation of alkenes to epoxides^42,43^. So far, many non-noble metal catalysts have been developed for catalyzing the reactions, but the harsh reaction conditions hinder their wide applications^44,45^. Therefore, conventional noble metal catalysts are still the most commonly employed catalysts in those reactions, but they generally suffer from the low efficiency of atom utilization and the inevitably high cost^43–46^. We thereby expect that atomically monodispersed heterogeneous catalysts can play a role in the reactions.

Herein, we have successfully synthesized a Pt_2_/mpg-C_3_N_4_ catalyst by using a simple wet-chemical method. The as-prepared sample possessed a dual-atom Pt_2_ structure that was evidenced with aberration correction transmission electron microscopy (TEM), X-ray absorption fine structure data, and first-principles simulations. The dual-atom Pt species exhibited excellent catalytic performance toward the selective hydrogenation of nitrobenzene to aniline, and behaved much better than the corresponding Pt single-atom catalysts and Pt nanoparticles (~2 nm). First-principles calculations revealed that the unique catalytic properties of Pt_2_/mpg-C_3_N_4_ originate from the easily breaking of the H–H bond in the H_2_ reactant and the effective release of the aniline product. The application of the superior Pt_2_/mpg-C_3_N_4_ catalyst has also been extended to the selective hydrogenation of benzaldehyde to benzyl alcohol and the epoxidation of styrene to styrene oxide, demonstrating the versatility of the dual-atom Pt species in heterogeneous catalysis.

## Results

### Synthesis and characterization of Pt_2_/mpg-C_3_N_4_ samples

The Pt_2_/mpg-C_3_N_4_ sample was synthesized using the wet-chemical strategy. (Ethylenediamine)iodoplatinum(II) dimer dinitrate and mesoporous graphitic carbon nitride (mpg-C_3_N_4_) were selected as the dual-atomic Pt precursor and the substrate. They were mixed and further pyrolyzed to remove the ligands from the dual-atom Pt precursor. The Pt_1_/mpg-C_3_N_4_ and the Pt nanoparticle/mpg-C_3_N_4_ samples were synthesized using the same method, except that the Pt species had been replaced by H_2_PtCl_6_ and Pt nanoparticles, respectively (see “Methods” for more details).

**Fig. 1:**
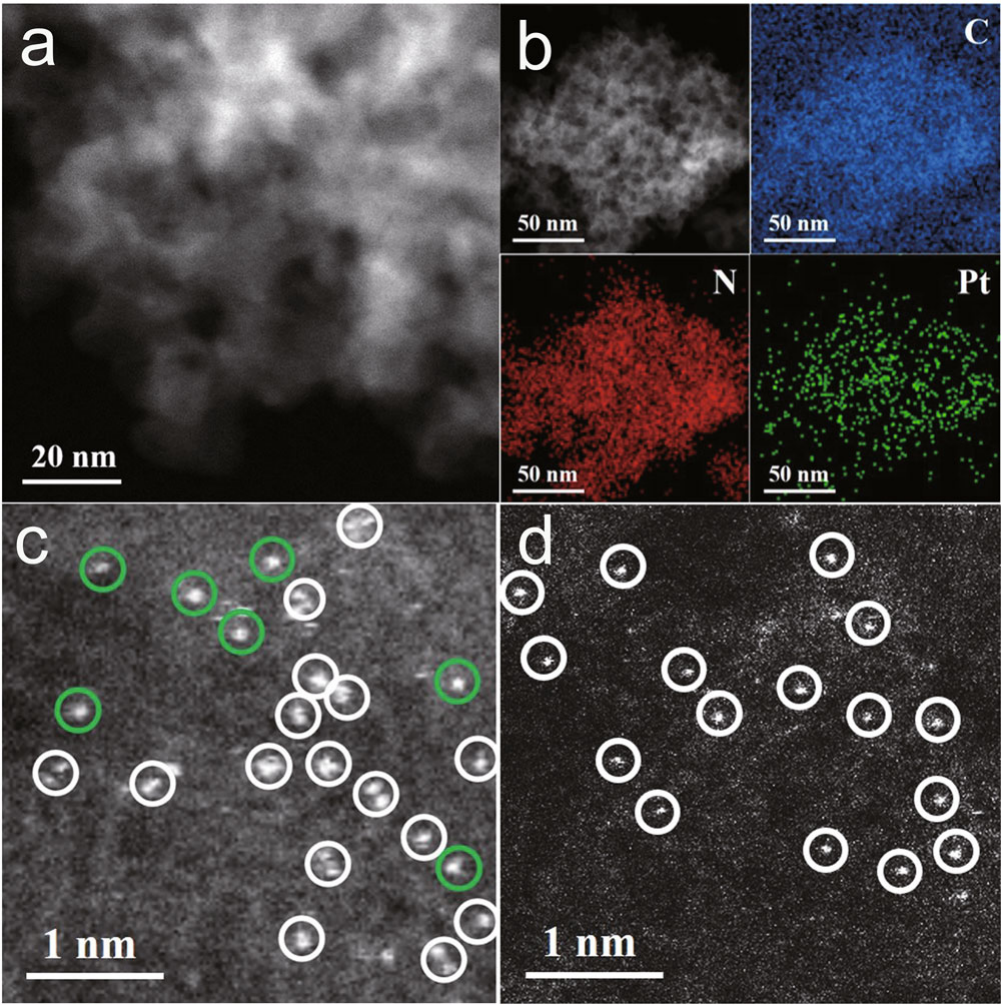
Characterization of Pt_2_/mpg-C_3_N_4_ sample. **a** HAADF-STEM image of Pt_2_/mpg-C_3_N_4_. **b** EDX mapping distributions of the C (blue), N (red), and Pt (green) elements, respectively. **c**, **d** AC HAADF-STEM images of the Pt_2_/mpg-C_3_N_4_ and Pt_1_/mpg-C_3_N_4_ samples, respectively.

The X-ray diffraction (XRD) pattern (Supplementary Fig. 1) demonstrated that the synthesized mpg-C_3_N_4_ sample has a graphitic packing structure^47,48^, and the disordered spherical pores of mpg-C_3_N_4_ were captured by the TEM image (Supplementary Fig. 2). Upon the loading of the dual-atom Pt precursor, neither Pt nanoparticles nor nanoclusters were observed in the TEM (Supplementary Fig. 3) and high-angle annular dark field scanning transmission electron microscopy (HAADF-STEM) images (Fig. 1a). Moreover, no additional diffraction peak of the Pt lattices was found in the XRD pattern (Supplementary Fig. 1). The energy-dispersive X-ray (EDX) spectroscopy further demonstrated a homogeneous distribution of the Pt species (Fig. 1b). The results from the inductively coupled plasma optical emission spectrometry estimated that the content of Pt is ~0.15 wt%. All the above results indicated that dual-atom Pt had been homogeneously dispersed on the mpg-C_3_N_4_ substrate. After the pyrolysis procedure, the infrared absorption peaks that correspond to the ligands of the precursor (at ~580, 825, 1005, 1050, 1340, 3090, and 3230 cm^−1^) were not observed in the Pt_2_/mpg-C_3_N_4_ sample, which supported a complete removal of the ligand molecules (Supplementary Fig. 4). To further confirm the dual-atomic feature of the Pt species, aberration-corrected (AC) HAADF-STEM was applied to characterize the Pt_2_/mpg-C_3_N_4_ sample. Many paired bright dots (marked with white circles) were observed in the AC HAADF-STEM image, which is consistent with the feature of two Pt atoms (Fig. 1c). Here, depending on the orientation of the Pt–Pt bond relative to that of the incident beam direction, the appearance of the paired bright dots can be different from each other (Supplementary Fig. 5), because the AC HAADF-STEM imaging only represents a two-dimensional projection of the three-dimensional Pt_2_/mpg-C_3_N_4_ samples^49^. Besides, a few isolated bright dots (marked with green circles) were also observed, which we attributed to an overlap of the two Pt atoms in the incident beam direction, or, to the incomplete imaging of the dual-atom Pt species owing to an incomplete focusing (Fig. 1c). To confirm the existence of the latter factor, we compared the different images that were collected within the same region of the sample, but with the focus of the imaging constantly changed (Supplementary Fig. 6). It can be seen that under different focusing conditions, many isolated dots can indeed be imaged as paired dots, demonstrating that the images actually come from the dual-atom Pt species. By contrast, the Pt_1_/mpg-C_3_N_4_ sample always exhibits and maintains the characteristics of a single bright spot (except under special circumstances as presented in Supplementary Fig. 11), further confirming the sharp difference between the dual-atom and the single-atom Pt species (Fig. 1d and Supplementary Figs. 7–10).

**Fig. 2:**
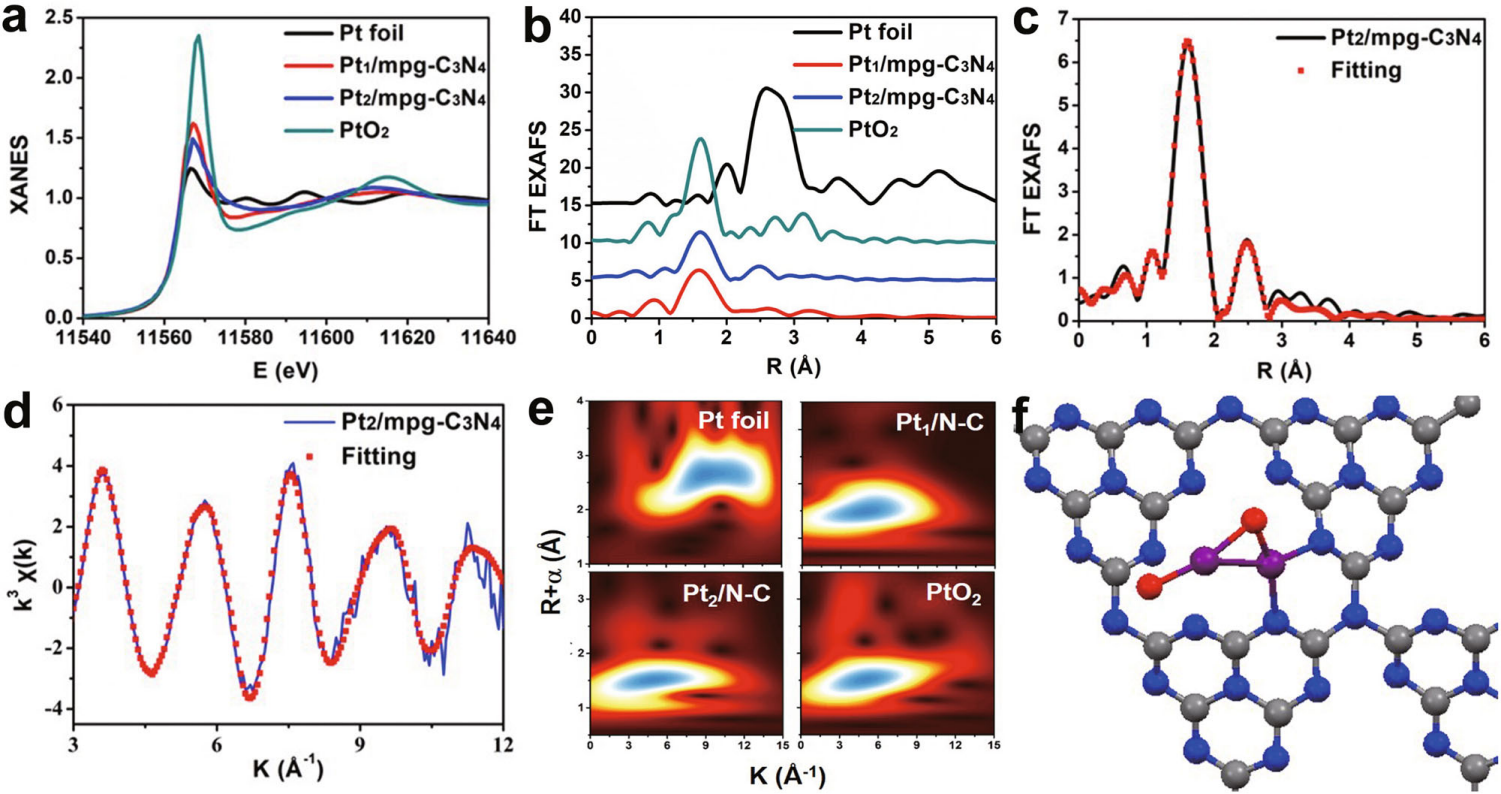
Pt *L*_3_-edge XAFS analysis. **a**, **b** XANES and FT EXAFS spectra of Pt_2_/mpg-C_3_N_4_, Pt_1_/mpg-C_3_N_4_, and corresponding references. **c**, **d** The FT EXAFS fitting spectrum of Pt_2_/mpg-C_3_N_4_ at *R*- and *k*-space, respectively. **e** WT EXAFS of Pt foil, Pt_1_/mpg-C_3_N_4_, Pt_2_/mpg-C_3_N_4_, and PtO_2_. **f** The schematic model of Pt_2_/mpg-C_3_N_4_ (C: gray; N: blue; O: red; Pt: purple).

X-ray absorption fine structure spectroscopy, which is a powerful technique for determining the chemical state and coordinated environment, was also applied to characterize the Pt species. The Pt *L*_3_-edge X-ray absorption near-edge structure (XANES) spectra of the Pt_2_/mpg-C_3_N_4_ and Pt_1_/mpg-C_3_N_4_ samples, as well as the corresponding references, are shown in Fig. 2a. Here, the white line intensity peak of dual-atom Pt_2_ is located between those of Pt foil and PtO_2_, indicating that the two Pt atoms possess positive charges. This can be attributed to the strong interaction between the dual-atom Pt species and the mpg-C_3_N_4_ substrate or partial oxidation of dual-atom Pt by the O_2_, which is similar to the case of Pt_1_/mpg-C_3_N_4_. The Fourier-transformed (FT) *k*^3^-weighted extended X-ray absorption fine structure (EXAFS) spectra of Pt_2_/mpg-C_3_N_4_ showed a sharp peak located at 1.62 Å, which is also similar to the result of Pt_1_/mpg-C_3_N_4_ and can be assigned to the Pt–N/O contributions (Fig. 2b). For Pt_2_/mpg-C_3_N_4_, another distinct peak at 2.44 Å was found, similar to the Pt–Pt path of Pt foil, but not observed in the spectrum of the Pt_1_/mpg-C_3_N_4_ sample. It reveals that the Pt–Pt path should also be taken into account in the spectrum of Pt_2_/mpg-C_3_N_4_. The wavelet transforms (WT) EXAFS analysis, which can discern scattering atoms and provide both *R*-space and *k*-space resolutions, was also employed (Fig. 2e). Here, the WT EXAFS spectrum of the Pt_2_/mpg-C_3_N_4_ sample showed a maximum at 4.6 Å^−1^ in *k*-space and at 1.6 Å in *R*-space, which we attributed to the Pt–O/N bonds. Besides, there were two distinct peaks at ~2.5 Å in *R*-space (*k* = 8 and 3.7 Å^−1^, respectively), which came from the contribution of the Pt–Pt bond. Especially, the peak at *k* = 3.7 Å^−1^ means that there were oxygen atoms connecting with Pt. According to these results, the structure of Pt_2_ consists of a Pt–Pt bond with surrounding O attached. The EXAFS fitting results further showed that the first peak at 1.62 Å comes from the Pt–N/O contributions and the second one at 2.44 Å is from the Pt–Pt path (Fig. 2c, d, Supplementary Figs. 12 and 13, and Supplementary Table 1). The coordination number of the Pt–N/O path was estimated to be 2.4 at the distances of 2.02 Å, and the coordination number from the second sphere by the Pt–Pt path was assessed to 1.1, with the corresponding distance being 2.61 Å.

The configuration of the Pt_2_/mpg-C_3_N_4_ sample was also explored by extensive first-principles calculations, with the optimized geometry shown Fig. 2f. As shown in Supplementary Fig. 14, the graphitic carbon nitride (g-C_3_N_4_) substrate is distorted showing obvious undulations. We first considered various kinds of Pt_2_/g-C_3_N_4_ structures without oxygen atoms (Supplementary Fig. 15) in the simulations, but none of them matched the XAFS information. It is worth noting that, since the measurements of the XAFS spectra were performed in air, the oxygen molecules contained in the atmosphere had interacted with and attached to the Pt species, which is also consistent with the WT EXAFS analysis result (Fig. 2e). We note in passing that such oxygen atoms will be removed by hydrogen molecules at the initial stage of the hydrogenation reactions discussed in the next section. We then explored the possible structures of dual-atom Pt_2_ with oxygen attached (Supplementary Fig. 16) and found one configuration (Fig. 2f) that agrees well with the results from the XAFS data. Here, the Pt–N/O and the Pt–Pt bond lengths were calculated to be 1.96 ± 0.14 and 2.55 Å, with the corresponding coordination numbers being 2.5 and 1.0, respectively, based on the model in Fig. 2f.

### Hydrogenation of nitrobenzene to aniline

**Fig. 3:**
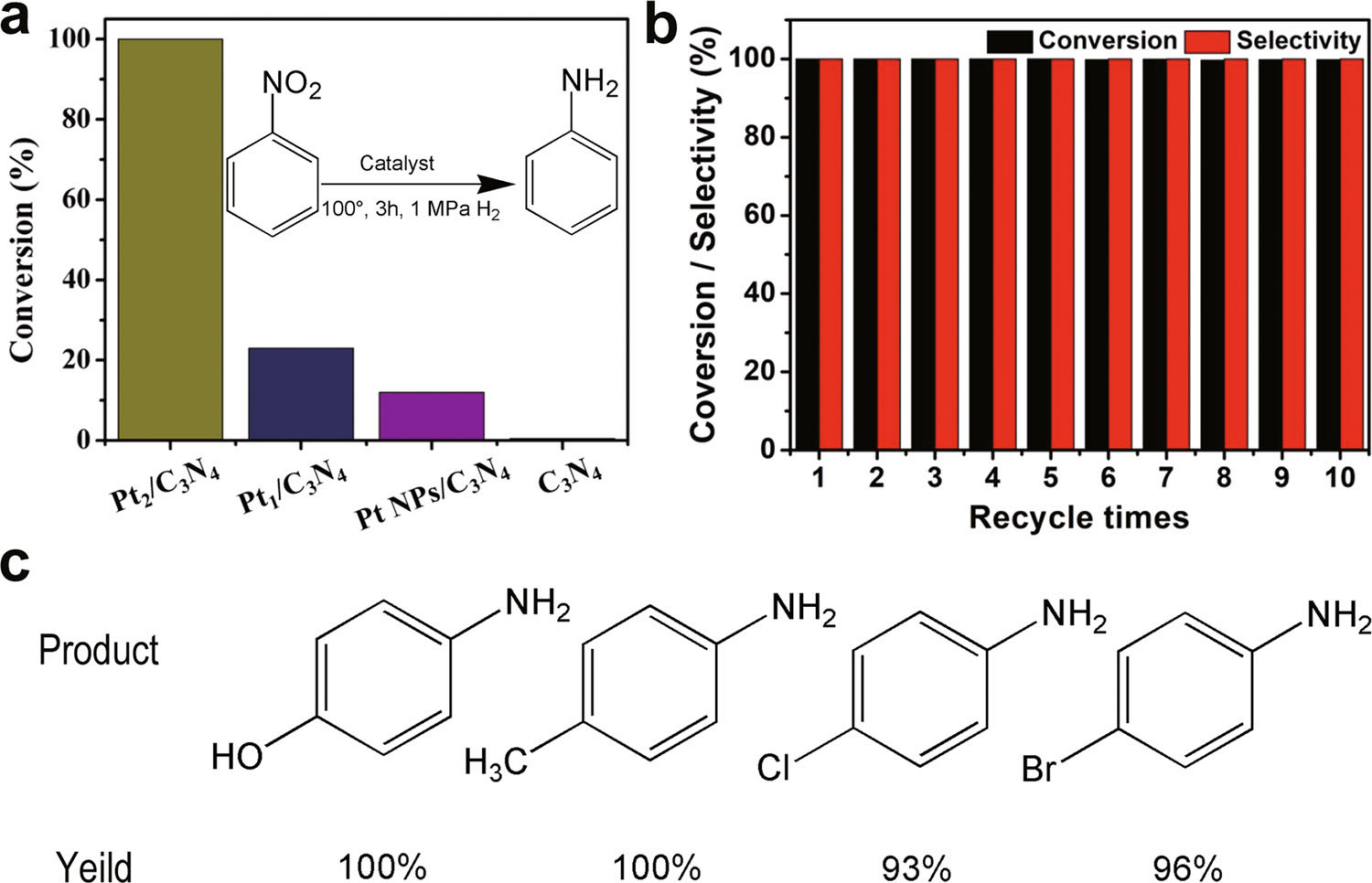
Hydrogenation of nitrobenzene. **a** Catalytic performance for the hydrogenation of nitrobenzene by Pt_2_/mpg-C_3_N_4_ and other reference samples. **b** Recycling of Pt_2_/mpg-C_3_N_4_ for the catalytic hydrogenation of nitrobenzene. **c** Hydrogenation of functionalized nitroarenes catalyzed by Pt_2_/mpg-C_3_N_4_.

The catalytic performance of Pt_2_/mpg-C_3_N_4_, Pt_1_/mpg-C_3_N_4_, and Pt nanoparticles (~2 nm)/mpg-C_3_N_4_ (Supplementary Fig. 17) were then investigated. Under the conditions of 1 MPa H_2_ and 3 MPa N_2_ pressure at 100 °C, a conversion of ˃99% was obtained on the Pt_2_/mpg-C_3_N_4_ catalyst for the hydrogenation of nitrobenzene to aniline, while no by-product was detected (Fig. 3a). Such Pt_2_/mpg-C_3_N_4_ sample can be reused at least ten times without any loss of the activity (Fig. 3b). After ten cycles, the AC HAADF-STEM image and the EXAFS spectrum of the Pt_2_/mpg-C_3_N_4_ material did not exhibit any change, indicating that the Pt species were still well dispersed as dual-atom Pt pairs (Supplementary Figs. 18 and 19). It may be worth mentioning that the mpg-C_3_N_4_ support is reactively inert under the same conditions. The corresponding conversions of Pt_1_/mpg-C_3_N_4_ and Pt nanoparticles/mpg-C_3_N_4_, by contrast, sharply dropped to 23% and 12%, respectively, demonstrating the uniqueness of the dual-atom Pt species in the catalytic properties (Fig. 3a). After the reaction, the Pt species in Pt_1_/mpg-C_3_N_4_ was still well dispersed as single atoms (Supplementary Fig. 20), and the size of the Pt nanoparticles on mpg-C_3_N_4_ did not change either (Supplementary Fig. 21), indicating that both Pt_1_ and Pt nanoparticles were stable in the catalytic process. To investigate whether the outstanding catalytic performance of the dual-atom Pt_2_ catalyst is general in the hydrogenation of nitroarenes, we have also explored the hydrogenation of several other nitroarene derivatives, including *p*-nitrophenol, *p*-nitrotoluene, tetrachloro-nitrobenzene, and tetrabromonitrobenzene. We found that Pt_2_/mpg-C_3_N_4_ exhibited excellent yields toward all corresponding anilines (Fig. 3c and Supplementary Table 2).

The changes in the oxidation state and chemical bonding of the Pt_2_ species during the process of the nitrobenzene hydrogenation were also detected by the time-dependent XAFS (Supplementary Figs.  22 and 23). The Pt *L*_3_-edge in the XANES spectra of Pt_2_/mpg-C_3_N_4_ showed that the intensity of the white line peaks became lower during the reaction (Supplementary Fig. 22), meaning that the oxidation state of Pt was smaller than that in the initial state. It is not surprising because the oxygen atoms attached to Pt can be removed by the hydrogen molecules. Besides, the EXAFS spectra showed that the first shell peak shifted from 1.57 to 1.55 Å (Supplementary Fig. 23), indicating that shorter chemical bonds like that of Pt–H appeared in the reaction process. Herein, it may be worth mentioning that the EXAFS exhibits a high sensitivity to the bond length change between center metal atoms and neighboring atoms, even when the change is as small as 0.01 Å (ref. ^50^). Although the shift of the first shell peak during nitrobenzene hydrogenation is slight, it can indeed be monitored by EXAFS.

**Fig. 4:**
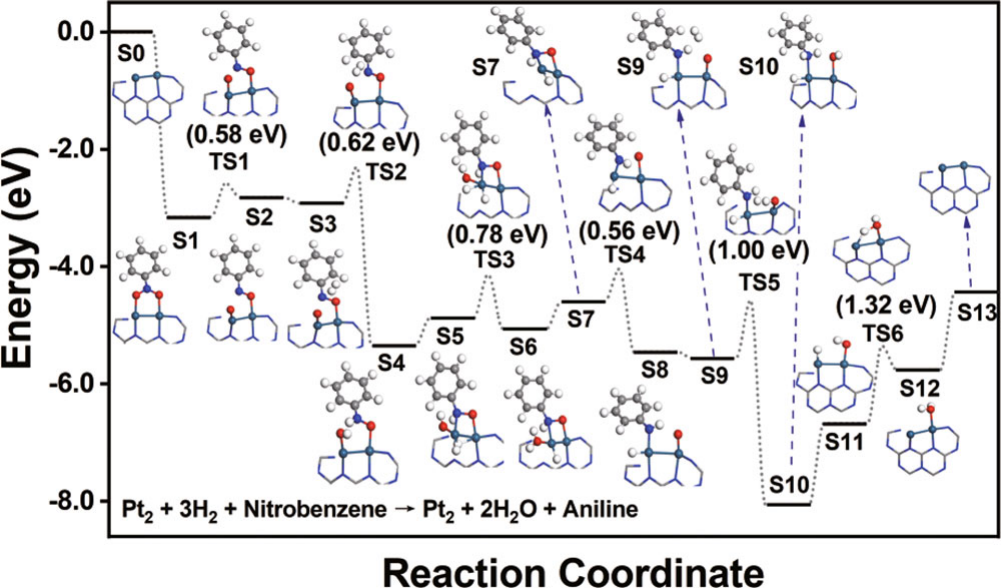
First-principles calculations of hydrogenation of nitrobenzene on Pt_2_/g-C_3_N4. Reaction pathway and computational energy profile of nitrobenzene hydrogenation on the Pt_2_/g-C_3_N_4_ catalyst. The label S0 represents the initial state and the subsequent labels S1–S12 represent a series of intermediate states. The labels TS1–TS6 (TS is short for transition states) represent a series of transition states. Here, only the key structures, i.e., the Pt_2_ catalytic system, as well as the adsorbate bound on it, are shown. The information regarding reactant molecules which have not been adsorbed, and/or product molecules which have been desorbed are labeled in the Supplementary Information (Supplementary Fig. 24). The teal, gray, blue, red, and white spheres represent the Pt, C, N, O, and H atoms, respectively.

To understand the underlying reason for the unique and excellent catalytic properties of the dual-atom Pt_2_ system, we have performed systematic first-principles simulations. It is found that the excellent and unique catalytic performance of the Pt_2_ species originates from the facile dissociation of the H_2_ reactant, which is induced by the diatomic characteristics of Pt, and the easy desorption of the aniline product. In Fig. 4 and Supplementary Fig. 24, we present the reaction pathway and the computational energy profile of the entire nitrobenzene hydrogenation process on the Pt_2_/g-C_3_N_4_ catalyst, which starts from the adsorption of the nitrobenzene reactant, and ends with the desorption of the aniline product and the regeneration of the Pt_2_ active site. As can be seen in Fig. 4, the nitrobenzene molecule connects with the two Pt atoms via the two oxygen atoms of the nitro group, showing an adsorption energy of −3.16 eV (a negative value means exothermic adsorption). Subsequently, one of the N–O bonds breaks (S1 → S2 via TS1; TS is short for transition state), and the corresponding energy barrier is only 0.58 eV. It is not surprising that the energy barrier is so low, since the N–O bonds have been well activated upon the nitrobenzene adsorption. In the Supplementary Fig. 26, we present the calculated electronic density of state (DOS) of Pt_2_/g-C_3_N_4_ upon the adsorption of nitrobenzene. One can see that the Pt diatomic species and the nitrobenzene adsorbate interact with each other at the Fermi level. By analyzing the corresponding spatial distribution, we find that the electronic state comes from the lowest unoccupied molecular orbital (LUMO) of nitrobenzene. Since the LUMO of nitrobenzene involves the antibonding interactions of the π orbitals between the N and the two O atoms (inset in the Supplementary Fig. 26), the facts that this LUMO appears at the Fermi level and is partially occupied upon nitrobenzene adsorption will inevitably bring about the weakening of the N–O bonds and the elongation of the corresponding bond lengths from 1.25 Å (the corresponding value of an isolated nitrobenzene molecule) to 1.35 Å, both of which lead to the easily breaking of the N–O bonds, and the generation of the unsaturated N and O adsorbates.

Interestingly, the unsaturated N and O adsorbates on each of the two Pt atoms can effectively promote the dissociation of the H_2_ reactant. In Fig. 4, one can see that after overcoming an energy barrier of only 0.62 eV (S3 → S4 via TS2), the first H_2_ molecule reacts with the N and the O atoms via the Eley–Rideal (ER) mechanism, producing the first N–H bond and a hydroxyl group. Here, the activation of the H_2_ molecule does not involve the participation of Pt atoms, reminiscent of the nitrogen-doped carbon nanotube arrays as metal-free electrocatalysts for the oxygen reduction reaction^51^. In the Supplementary Fig. 25, we present the calculated electronic DOS of the two H atoms that are involved in the H_2_ dissociation for the configurations S3 and TS2 in Fig. 4. One can see that while in S3 (left panel in the Supplementary Fig. 25), the two H atoms have the same electronic structure that is very close to that of the hydrogen atoms in an isolated H_2_ molecule, in TS2 (right panel in the Supplementary Fig. 25), the electronic structures of the two H atoms are significantly different. In particular, at the peaks of 0.39 and 0.09 eV below the Fermi level, the H atom close to the N atom (with the H–N distance being 1.87 Å) exhibits an obvious electronic state distribution, while the H atom close to O (with the H–O distance being 1.52 Å) has almost no distribution. Such remarkable contrast can also be visualized from the spatial distributions of the electronic states within the corresponding energy intervals (inset in the right panel). Here, one of the two H atoms as well as the unsaturated N and O adsorbates exhibits the distributions. The results indicate that the H_2_ activation is facilitated by the produced N and O atoms, and is promoted by a polarization effect induced by O. The latter is further supported by the Bader charge analysis, showing that in TS2, the two H atoms carry charges of +0.20 (close to O) and −0.08 (close to N), respectively.

From S5 to S6, the second H_2_ molecule adsorbs on one Pt atom and then dissociates after overcoming a barrier of 0.78 eV (TS3). Upon desorption of the produced water molecule (S6 to S7), the second N–O bond breaks in a similar way, showing an energy barrier of 0.56 eV (S7 → S8 via TS4). As the third H_2_ molecule participates in the reaction via the same ER mechanism, the second N–H bond forms and the aniline molecule is produced after overcoming an energy barrier of 1.00 eV (S9 → S10 via TS5). Here, the value of the energy barrier is higher than that when the first H_2_ molecule dissociated in the similar way (TS2). This may be due to the weakening of the interaction between the H_2_ reactant and the N atom, since the latter has already formed an N–H bond. What follows, the aniline product desorbs from the Pt_2_ system, which is facilitated by a change in the configuration of the hydrogen adsorbate on Pt (S10 → S11). Then, the remaining one hydrogen atom and one hydroxyl group on the Pt_2_ site form a water molecule through hydrogen transfer (S11 → S12 via TS6), and upon the desorption of water (S12 → S13), the catalyst restores. It may be worth noting that the process from S10 to S13 could take place more easily than what the energy profile reflects, since the transfer of the hydrogen atom will be promoted by the quantum tunneling effect, and the water desorption can be promoted by the competitive adsorption of the nitrobenzene reactant.

**Fig. 5:**
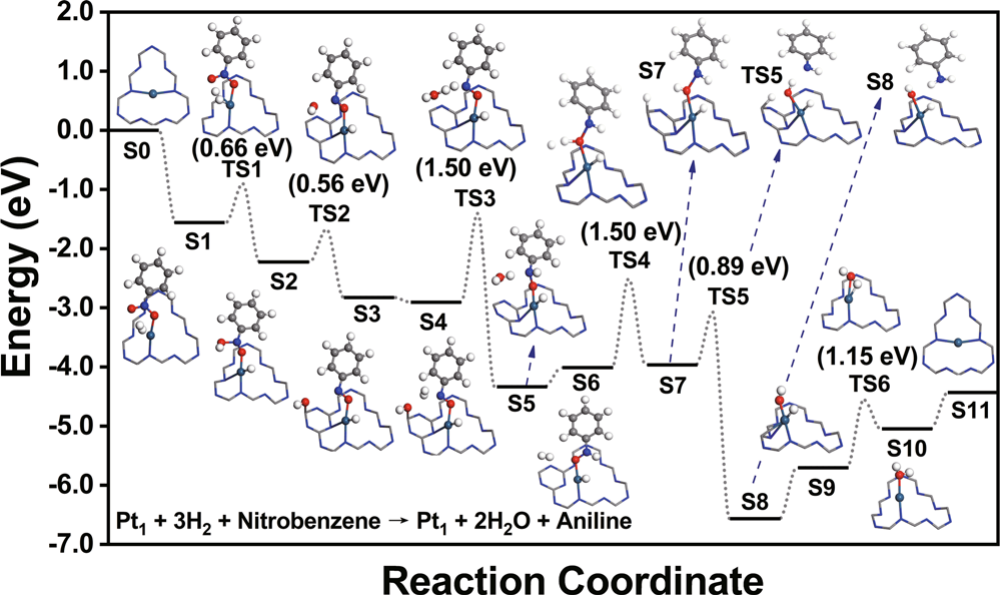
First-principles calculations of hydrogenation of nitrobenzene on Pt_1_/g-C_3_N4. Reaction pathway and computational energy profile of nitrobenzene hydrogenation on the Pt_1_/g-C_3_N_4_ system. The label S0 represents the initial state and the subsequent labels S1–S10 represent a series of intermediate states. The labels TS1–TS6 (TS is short for transition states) represent a series of transition states. Here, only the key structures, i.e., the Pt_1_ catalytic system as well as the adsorbate bound on it, are shown. The information regarding reactant molecules which have not been adsorbed, and/or product molecules which have been desorbed are labeled in the Supplementary Information (Supplementary Fig. 27). The teal, gray, blue, red, and white spheres represent the Pt, C, N, O, and H atoms, respectively.

As aforementioned, the underlying reason why Pt_2_ can exhibit the unique catalytic performance in the nitrobenzene hydrogenation, compared with the Pt single atoms and nanoparticles, is that not only the activation and dissociation of the H_2_ reactant can easily occur, the desorption of the generated aniline product is also a facile process. By contrast, however, the above two features are not simultaneously possessed in either Pt_1_ or the Pt nanoparticles. We first compare the difference between Pt_1_ and the dual-atom catalyst. In Fig. 5 and Supplementary Fig. 27, we present the reaction pathway and the computational energy profile of nitrobenzene hydrogenation on Pt_1_/g-C_3_N_4_. Since the Pt_1_/g-C_3_N_4_ system contains only one Pt atom, the unsaturated N and O atoms cannot be generated as in the case of Pt_2_. Fortunately, since the Pt atom directly participates in the dissociation of the first H_2_ molecule (S1 → S2 via TS1), the corresponding energy barrier is not very high, being only 0.66 eV. However, since neither Pt atoms nor the two unsaturated N and O atoms are present in the dissociation of the second (S4 → S5 via TS3) and the third (S6 → S7 via TS4) H_2_ molecules, the reaction energy barriers significantly increase, both of which reach 1.50 eV. Thus, it is not surprising that the Pt_1_ system cannot exhibit the same excellent catalytic properties as Pt_2_.

Regarding the Pt nanoparticles, it was found that the hydrogenation was induced and assisted by the adsorbed H atoms that were produced via a spillover process on the outermost layers of the nanoparticles (simulated using a Pt(111) surface in the simulations)^52^. The overall energy barrier was calculated to be only 0.75 eV (ref. ^50^), indicating that the occurrence of the hydrogenation is not difficult. The obstacle, however, comes from the desorption step of the aniline product. Our calculations show that the adsorption energy of aniline on Pt(111) is as high as −1.70 eV, and in the adsorption configuration (Supplementary Fig. 28A), several C atoms of the phenyl group are involved in the bonding with the surface Pt atoms. It means that upon desorption, the aniline molecule needs to overcome a high energy barrier. Furthermore, since the adsorption of nitrobenzene (showing an adsorption energy of −1.35 eV) is weaker than that of aniline on Pt(111), the desorption of the aniline product cannot be promoted via a competitive adsorption of the reactant. Such problems, however, do not appear in the Pt_2_ system. Despite that the adsorption energy of aniline on Pt_2_ is as high as −2.58 eV, the adsorption of the nitrobenzene reactant is stronger, showing an adsorption energy of −3.16 eV. The stronger interaction of the adsorption site with the nitrobenzene reactant is able to promote aniline desorption. Moreover, there is only one N–Pt bond connecting aniline with the Pt_2_ catalyst (Supplementary Fig. 28C), and the configuration change of the hydrogen adsorbate on Pt can also promote the desorption process (S10 → S11 in Fig. 4). Thus, the aniline desorption can be easily achieved and is no longer an obstacle on Pt_2_.

By comparing the reaction pathways in Figs. 4 and 5, one can see that for the Pt_1_/g-C_3_N_4_ system, the g-C_3_N_4_ framework not only serves as a substrate to anchor the Pt atoms, but also, its C atoms can directly participate in the nitrobenzene hydrogenation. For example, in the cleavage of the first N–O bond on Pt_1_/g-C_3_N_4_ (Fig. 5), an adjacent carbon atom to Pt acts as the adsorption site to stabilize the produced OH radical (S2 → S3 via TS2). In the activation and dissociation of the third H_2_ molecule (S6 → S7 via TS4), this carbon atom, together with the oxygen atom nearby, stabilize the two produced hydrogen atoms. The adsorbates on the carbon atom can also affect the electronic structure of this atom, as well as that of the entire g-C_3_N_4_ framework. In the Supplementary Fig. 29, we present the calculated electronic DOS of this C atom (left panel) and the g-C_3_N_4_ framework (right panel) for the configurations S2 (before OH connects to the C atom), S3 (with OH bound to the C atom), and S5 (after OH leaves the C atom), as shown in Fig. 5. One can see that when the OH group does not form a bond with the C atom, no matter the system adopts the configuration S2 or S5, the electronic structures of both the C atom and the g-C_3_N_4_ framework are not much different. However, when the OH radical is attached to the C atom, the electronic structures undergo obvious changes: for the C atom that is bound to OH, the unoccupied electronic states within 3 eV above the Fermi level disappear; while for the g-C_3_N_4_ framework, the entire DOS undergoes a right shift relative to the Fermi level. The changes in the above electronic structures also shows that the OH group has a strong interaction with the C atom, further supporting that OH is a radical rather than an anion.

### Hydrogenation of benzaldehyde and epoxidation of alkenes

**Fig. 6:**
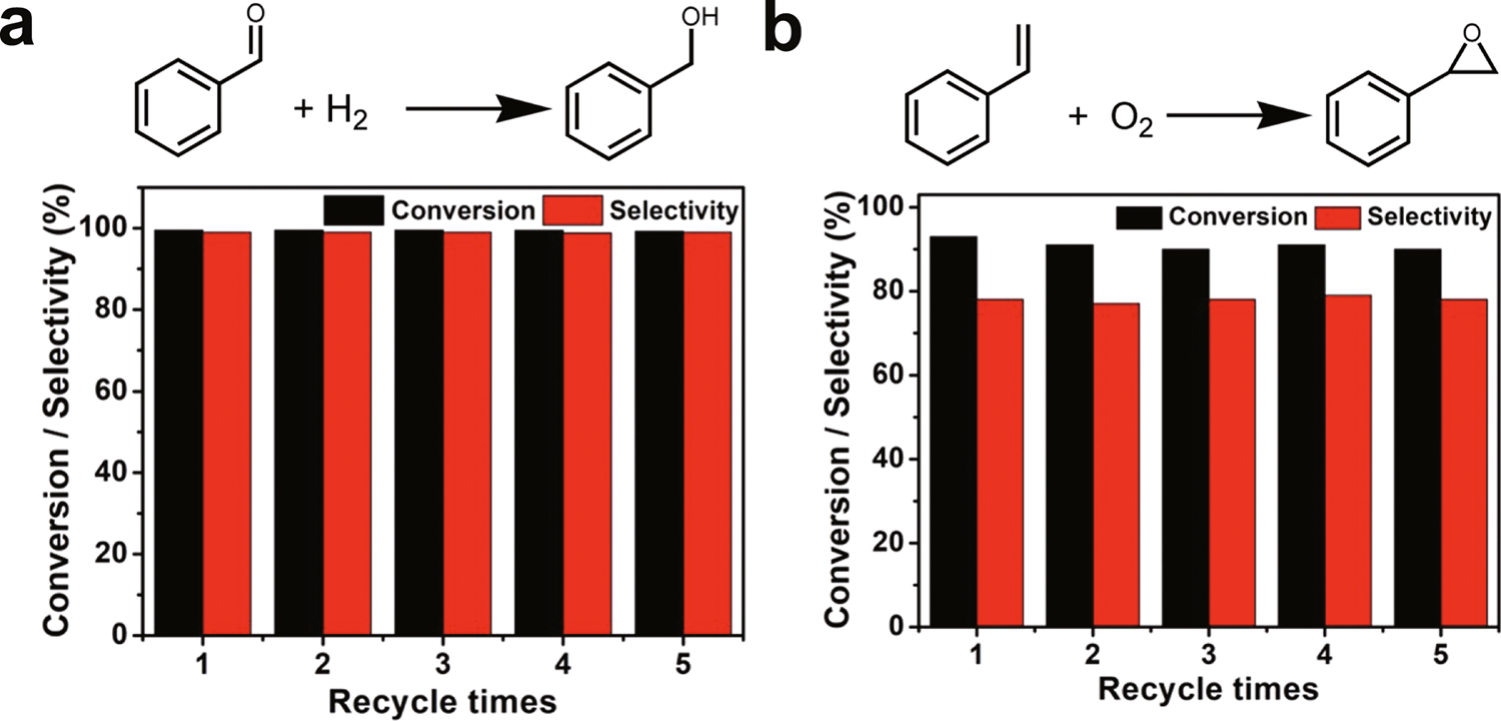
Hydrogenation of benzaldehyde and epoxidation of styrene. **a** Catalytic performance for the hydrogenation of benzaldehyde by using the Pt_2_/mpg-C_3_N_4_ catalyst. **b** Corresponding catalytic performance for the epoxidation of styrene.

The produced Pt_2_/mpg-C_3_N_4_ catalyst is versatile and can be employed in other important reactions besides the selective hydrogenation of nitrobenzene. For example, under the conditions of 8 MPa of an H_2_ and N_2_ mixture (1:1) at 120 °C, the Pt_2_/mpg-C_3_N_4_ sample showed optimal catalytic performance toward the hydrogenation of benzaldehyde to benzyl alcohol (Fig. 6a). Specifically, ˃99% conversion and ˃99% selectivity were achieved for 7 h. Moreover, the catalyst can be reused at least five times without obvious loss of the activity (Fig. 6a). In Supplementary Figs. 30 and 31, we display the reaction pathway and the computational energy profile of benzaldehyde hydrogenation on the Pt_2_/g-C_3_N_4_ catalyst, showing that the Pt_2_ species can effectively catalyze the hydrogenation of benzaldehyde toward benzyl alcohol. Especially, the H_2_ dissociation can be promoted by the Pt_2_ species, and the C=O bond can also be activated by Pt_2_ (S1), as reflected in the elongation of the corresponding bond length from 1.23 Å (the value of an isolated benzaldehyde molecule) to 1.40 Å. In addition, the desorption of benzyl alcohol can also be promoted by the configuration change of the hydrogen adsorbate (S5 → S6). These are similar to the reaction pattern of the aforementioned nitrobenzene hydrogenation, demonstrating the unique role of the dual-atom species in the catalytic hydrogenation reactions.

**Fig. 7:**
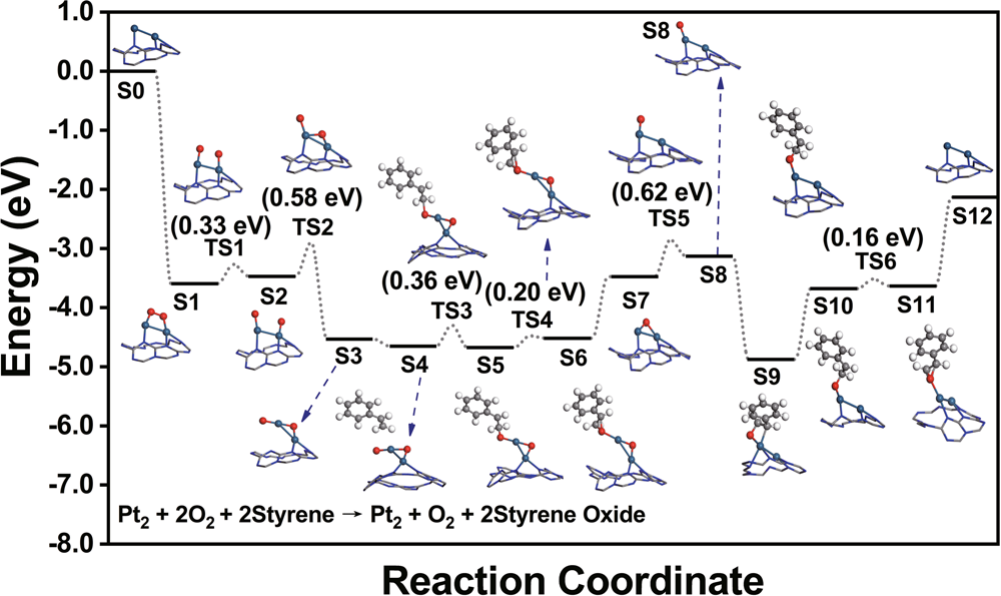
First-principles calculations of styrene epoxidation on Pt_2_/g-C_3_N_4_. Reaction pathway and computational energy profile of styrene epoxidation on the Pt_2_/g-C_3_N_4_ catalyst. The label S0 represents the initial state and the subsequent labels S1 – S11 represent a series of intermediate states. The labels TS1 – TS6 (TS is short for transition states) represent a series of transition states. Here, only the key structures, i.e., the Pt_2_ catalytic system as well as the adsorbate bound on it, are shown. The information regarding reactant molecules which have not been adsorbed, and/or product molecules which have been desorbed are labeled in the Supplementary Information (Supplementary Fig. 32). The teal, gray, blue, red, and white spheres represent the Pt, C, N, O, and H atoms, respectively.

As another type of significantly important reactions^53^, the epoxidation of alkenes in liquid relies on extensive use of expensive oxidants or co-reagents, which leads to a large increase in the cost. Our prepared Pt_2_/mpg-C_3_N_4_ catalyst exhibited excellent catalytic performance toward the epoxidation of styrene when only O_2_ molecules were used as the oxidant, which well circumvents the above problem. In Fig. 6b, one can see that Pt_2_/mpg-C_3_N_4_ exhibited a conversion of 93% and a selectivity of 78% after 12 h, and this is one of the best results for the epoxidation of styrene^54,55^. Moreover, the Pt_2_/mpg-C_3_N_4_ catalyst can be reused at least five times without obvious loss of the activity (Fig. 6b). To understand the detailed process of the reaction, in Fig. 7 and Supplementary Fig. 32, we display the corresponding pathway and the energy profile of the styrene epoxidation. It should be noted that since the reaction is carried out in an O_2_ atmosphere, the active site is no longer the isolated dual Pt atoms as in the cases of the nitrobenzene and benzaldehyde hydrogenations. Here, an O_2_ molecule first adsorbs and then transforms into two oxygen atoms by overcoming an energy barrier of 0.58 eV (S0 → S3 via TS1 and TS2). The generated Pt_2_O_2_ species (S3) contains a one-coordinated oxygen atom and a two-coordinated oxygen atom, consistent with what we have observed in the EXAFS measurements. The Pt_2_O_2_ species, in fact, is the true active site of the styrene epoxidation. In the next few steps (from S4 to S6), the one-coordinated oxygen atom interacts with the C=C bond of` the alkene and converts styrene to the corresponding epoxide. As the first styrene oxide molecule desorbs, there is only a two-coordinated oxygen atom locating at the Pt_2_ site (S7). Such type of oxygen can be converted to a new one-coordinated oxygen atom by crossing an energy barrier of 0.62 eV (S7 → S8 via TS5), and then, participates in the next few steps of the epoxidation reaction (from S9 to S11). Upon the desorption of the second styrene oxide molecule, as well as the adsorption of another O_2_ molecule, the catalytic reaction cycle starts again. In Fig. 7, one can see that the one-coordinated oxygen atoms can directly participate in the epoxidation reaction, while the two-coordinated oxygen atom is not involved in the reaction until it is converted to the one-coordinated configuration. It is not surprising because the one-coordinated oxygen atom connects with Pt_2_ via only one chemical bond and is thus less bound, which makes it easier to participate in the reaction and be grabbed by the styrene molecules. This phenomenon is very similar to the case of the diatomic Fe_2_ system, which we used as dual-atom catalyst in the epoxidation of *trans*-stilbene molecules^28^.

## Discussion

In conclusion, we have reported an atomically monodispersed dual-atom Pt heterogeneous catalyst, which exhibited excellent catalytic properties in selective hydrogenation and epoxidation reactions. Multiple characterization techniques, including AC STEM, XAFS spectra, and first-principles simulations, were employed to capture the structural and chemical nature of the dual-atom Pt species. Compared with the mpg-C_3_N_4_-supported Pt single-atom catalysts and ultra-small Pt nanoparticles (~2 nm), Pt_2_/mpg-C_3_N_4_ exhibited much better performance toward the selective hydrogenation of nitrobenzene, due to the facile dissociation of the H_2_ reactant and the effective release of the aniline product. More interestingly, the prepared Pt_2_/mpg-C_3_N_4_ catalyst is versatile and can be applied in catalyzing other important reactions, like the hydrogenation of aldehyde compounds and the epoxidation of alkenes.

## Methods

### Materials

Cyanamide (98%), LUDOXR AS-40 colloidal silica 40 wt% suspension in H_2_O, ammonium hydrogen difluoride, hydrogen hexachloroplatinate(IV) hydrate, platinum(II) 2,4-pentanedionate, and (ethylenediamine)iodoplatinum(II) dimer dinitrate were purchased from Innochem. Ethanol, *N*,*N*-dimethylformamide, and *n*-hexane were purchased from Sinopharm Chemical Reagent Co. Ltd. Oleylamine and borane-*tert*-butylamine were purchased from Sigma-Aldrich Reagent Company.

### Methods

#### Synthesis of mpg-C_3_N_4_, and Pt nanoparticles

The synthesis of mpg-C_3_N_4_ was according to the previous method without any change^46^. In the synthesis of the Pt nanoparticles, 20 mg Pt(II) acetylacetonate was dissolved in 10 mL octadecenylamine (OAm) at 120 °C. A solution of 100 mg borane-*tert*-butylamine in 2 mL OAm was added quickly into the above solution. After 2 min, the flask was heated to 140 °C and then kept at 140 °C for an hour. After that, the solution was cooled to room temperature, followed by ethanol washing.

#### Synthesis of Pt_2_/mpg-C_3_N_4,_ Pt_1_/mpg-C_3_N_4_, and Pt nanoparticles/mpg-C_3_N_4_

In the typical synthesis of Pt_2_/mpg-C_3_N_4_, 500 mg mpg-C_3_N_4_ and 5 mg (ethylenediamine)iodoplatinum(II) dimer dinitrate were dissolved in 100 mL DMF under the stirring condition. After continuous stirring for ~12 h, the resulting product was centrifuged and the supernatant was discarded. The obtained precipitate was washed with DMF and methanol. The as-prepared powder was treated under the N_2_ atmosphere at 300 °C for 2 h. The loading of Pt, determined by inductively coupled plasma optical emission spectrometer (ICP-OES) analysis, was 0.15 wt%. Thermogravimetric analysis showed no weight loss at 300 °C, indicating that the ligands were removed completely (Supplementary Fig. 33). The synthesis methods of Pt_1_/mpg-C_3_N_4_ and Pt nanoparticles/mpg-C_3_N_4_ are similar to that of Pt_2_/mpg-C_3_N_4_ with some modifications. In the synthesis of Pt_1_/mpg-C_3_N_4_, 500 mg mpg-C_3_N_4_ was dissolved in 100 mL H_2_O. Then, a solution of 2.5 mg H_2_PtCl_6_ in 10 mL H_2_O was added to the above solution under vigorous stirring. After continuous stirring for ~12 h, the resulting product was centrifuged and the supernatant was discarded. The as-prepared powder was treated under the N_2_ atmosphere at 125 °C for 2 h. The loading of Pt, determined by ICP-OES analysis, is 0.18 wt%. In the synthesis of Pt nanoparticles/mpg-C_3_N_4_, 5 mg Pt nanoparticles and 500 mg mpg-C_3_N_4_ were dissolved in a mix solvent of 200 mL ethanol and *n*-hexane (1:1 v/v) under stirring at room temperature for 12 h. The product was separated by centrifugation, then washed with methanol. The as-prepared powder was treated under the N_2_ atmosphere at 125 °C for 2 h. The Pt loading is 0.42 wt% as determined by ICP-OES analysis.

### Catalytic tests

#### Typical procedure for the hydrogenation of nitrocompound

In the typical experiment, the reaction mixture containing 1 mmol nitrocompound (nitrobenzene, *p*-nitrophenol, *p*-nitrotoluene, tetrachloro-nitrobenzene, and tetrabromonitrobenzene), catalyst (equal 0.000373 mmol Pt for Pt_1_/mpg-C_3_N_4_, Pt_2_/mpg-C_3_N_4_, and Pt nanoparticles/mpg-C_3_N_4_ or 50 mg mpg-C_3_N_4_), and 10 mL isopropanol were loaded into the reactor. The reactor was sealed and pressurized with 1 MPa H_2_ and 3 MPa N_2_ to a setting point. The reaction was then heated to the 100 °C and kept for 3 h. The products were identified by gas chromatography–mass spectrometer (GC–MS) and gas chromatography (GC).

Following the hydrogenation reaction, the reaction mixture was centrifuged to recover the catalyst, which was first washed with acetone and then water, followed by drying under vacuum oven at 50 °C before being employed for the next catalytic test.

#### Typical procedure for the hydrogenation of benzaldehyde

In the typical experiment, the reaction mixture containing benzaldehyde (1 mmol), 50 mg Pt_2_/mpg-C_3_N_4_, and 10 mL isopropanol were loaded into the reactor. The reactor was sealed, purged three times with 1 MPa of N_2_ at and then pressurized at 8 MPa of an H_2_ and N_2_ mixture (1:1) to a setting point. The reaction was then heated to the 120 °C temperature and kept for 9 h.

#### Typical procedure for the epoxidation of styrene

A total of 1 mmol styrene, 20 mg Pt_2_/mpg-C_3_N_4_, and 5 mL 1,4-dioxane were mixed in a 20 mL Schleck tube. Then, the air in the Schleck tube was removed by an oil pump. An O_2_ balloon was used to blow ~1 atm O_2_ into the tube. Finally, the reaction vessel was heated in an oil bath at 100 °C for 12 h.

The products were identified by the gas chromatography–mass spectrometry (GC–MS, Thermo Fisher Scientific-TXQ Quntum XLS), and were quantitatively analyzed by the GC (Shimadzu, GC-2010 Plus), equipped with the flame ionization detector and a (30 m × 0.25 mm × 0.25 mm) KB-WAX capillary column (Kromat Corporation, USA), using *n*-octanol as the internal standard. Operation parameters for the GC–MS measurements: the inlet temperature was 250 °C, the MS transfer line temperature was 250 °C, and the ion source temperature was 280 °C. The column temperature was first kept at 40 °C for 1 min, and then raised to 150 °C with a ramp rate of 20 °C min^−1^, and was later raised to 250 °C with a ramp rate of 15 °C min^−1^. Finally, it was kept at 250 °C for 7 min. Operation parameters for the GC measurements: the vaporization temperature and the detector temperature were both 270 °C. The column temperature was first kept at 40 °C for 1 min, and then raised to 240 °C with a ramp rate of 10 °C min^−1^ and was finally kept at 240 °C for 6 min.

### Characterization

XAFS measurements and analysis. The XAFS data of Pt *L*_3_-edge was collected in fluorescence excitation mode using a Lytle detector at 1W1B station in Beijing Synchrotron Radiation Facility. The EXAFS data were processed by the standard procedures using the ATHENA module implemented in the IFEFFIT software packages. To obtain the quantitative structural parameters around central atoms, the least-squares curve parameter fitting was performed, using the ARTEMIS module of IFEFFIT software packages.

### Details of calculations

Most of the calculations were conducted using the Vienna ab initio simulation package^56,57^. The projector augmented wave approach^58^ was employed, and the energy cutoff of the plane-wave basis set was set to 500 eV. The exchange–correlation interactions were described by the optPBE-vdW functional^59,60^, which explicitly includes effects of van der Waals forces. The first Brillouin zone was sampled using a 3 × 3 × 1 Monkhorst-Pack grid^61^. To simulate the mpg-C_3_N_4_ and the Pt nanoparticles, a g-C_3_N_4_ monolayer and a Pt(111) slab model (four layers with 16 Pt atoms within each layer) were employed, respectively. Structural relaxations were performed until the maximum residual force on each atom was <0.03 eV Å^−1^. Transition states were located using the climbing-image nudged elastic band method^62^ with a force criterion of 0.10 eV Å^−1^. Each transition state has been confirmed via the vibrational mode analysis to ensure that it is indeed connected to the correct initial and final states of the elementary reaction step. The corresponding imaginary frequencies were listed in Supplementary Tables 3–6 and the raw file (.xyz) for the associated vibirational modes were uploaded to the NoMaD repository (see the “Data availability” section). The LUMO orbital of the isolated nitrobenzene molecule is calculated with the Gaussian 16 package^63^ using the PBE^64^ functional and the 6-31 G(d) basis set. All structures were visualized using the program VESTA^65^.

## Supplementary information

Supplementary Information

Peer Review File

## Data Availability

The computational data for Figs. 4, 5, and  7, and Supplementary Fig. 30 is available in the NoMaD Repository (http://nomad-repository.eu/) via 10.17172/NOMAD/2021.04.28-1. And all relevant data that support the findings of this study are available from the authors upon reasonable request.
