# Supplementary material for: Dual-atom Pt heterogeneous catalyst with excellent catalytic performances for the selective hydrogenation and epoxidation
Source: Nat Commun. 2021 May 26;12:3181. doi: 10.1038/s41467-021-23517-x (PMC8155026; doi:10.1038/s41467-021-23517-x)
Supplement: Supplementary file 1 — Supplementary Information [file 41467_2021_23517_MOESM1_ESM.pdf]

Dual-atom Pt heterogeneous catalyst with excellent catalytic performances for the selective hydrogenation and epoxidation

Supplementary Information

Tian et al.

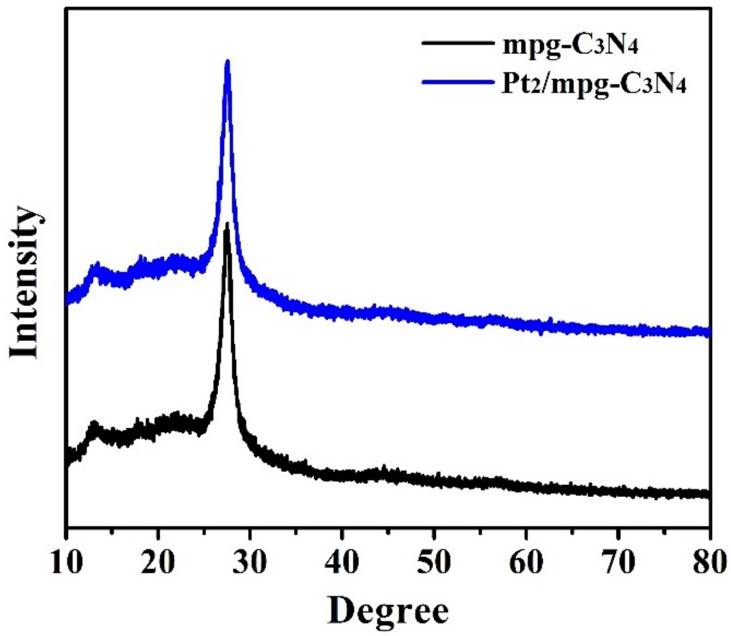

Supplementary Figure 1. XRD patterns of the as-prepared mpg-C_3N_4 and Pt_2/mpg-C_3N_4.

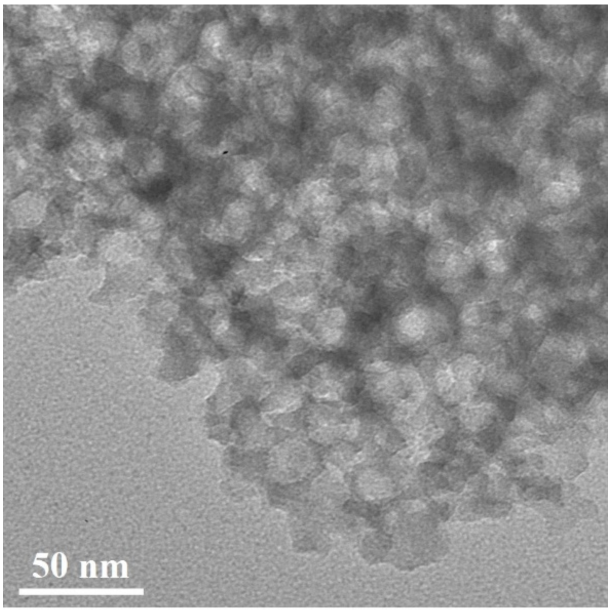

Supplementary Figure 2. TEM image of mpg-C_3N_4 sample.

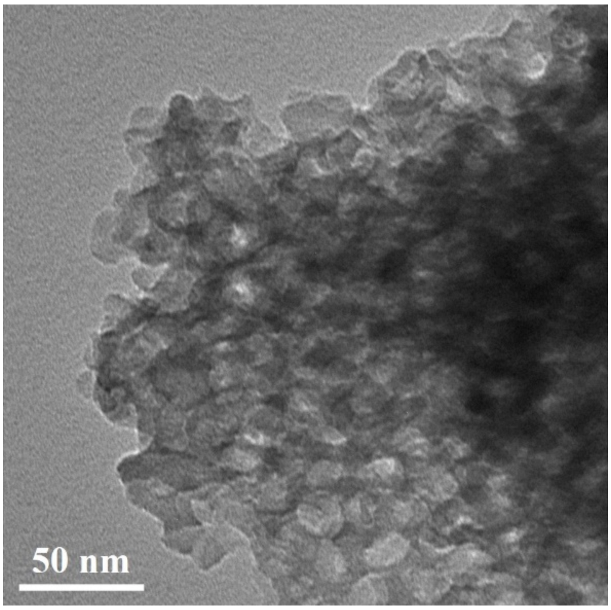

Supplementary Figure 3. TEM image of Pt_2/mpg-C_3N_4 before the reaction.

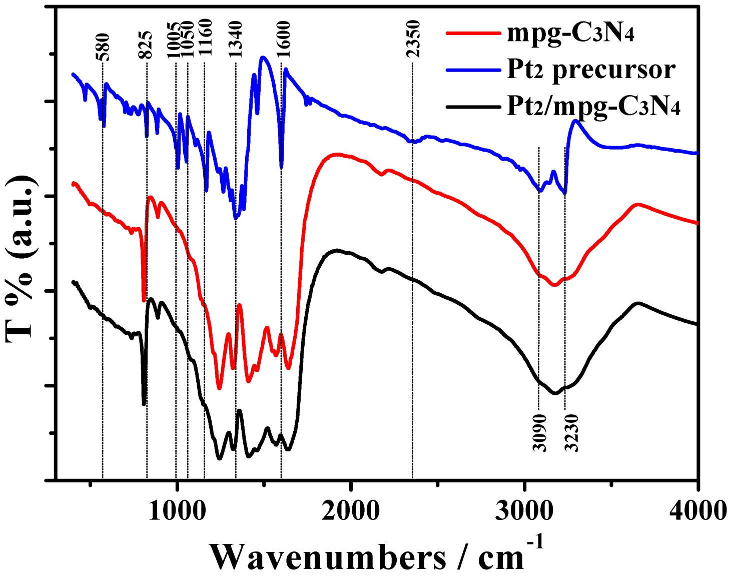

Supplementary Figure 4. IR patterns of the mpg-C_3N_4 (red), Pt_2 precursor (blue), and Pt_2/mpg-C_3N_4 (black).

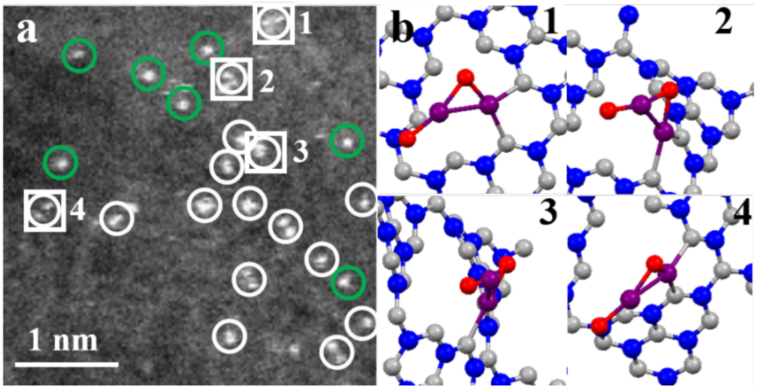

Supplementary Figure 5. (a) AC HAADF-STEM image of Pt_2/mpg-C_3N_4. (b) The corresponding proposed orientations of the sample for the different detailed features of Pt_2 in the areas 1, 2, 3, and 4.

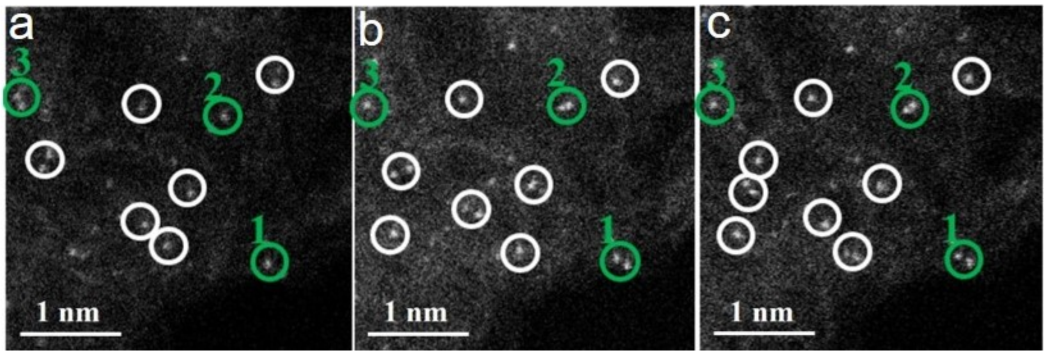

Supplementary Figure 6. AC HAADF-STEM images of Pt_2/mpg-C_3N_4 in the different focusing.

To confirm the existence and the consequence of the incomplete focusing "problem", we have compared the different images that were collected within the same region of the sample but with the focus of the imaging constantly changed (Supplementary Figure 6). It can be seen that under different focusing conditions, isolated dots can indeed be imaged as paired dots. For example, with the focusing condition changed, the two isolated bright dots in the areas marked as "1" and "2" in the Supplementary Figure 6a were imaged as paired bright dots in the Supplementary Figures 6b and S6c. Meanwhile, the area "3" where an isolated dot was imaged in the Supplementary Figs. S6b and S6c can also exhibit paired bright dots in the Supplementary Figure 6a. Considering that the Pt_1/mpg-C_3N_4 sample hardly exhibited and cannot maintain the feature of paired bright dots, the above results clearly demonstrate that the reported images in Fig. 1c actually come from the dual-atom Pt species.

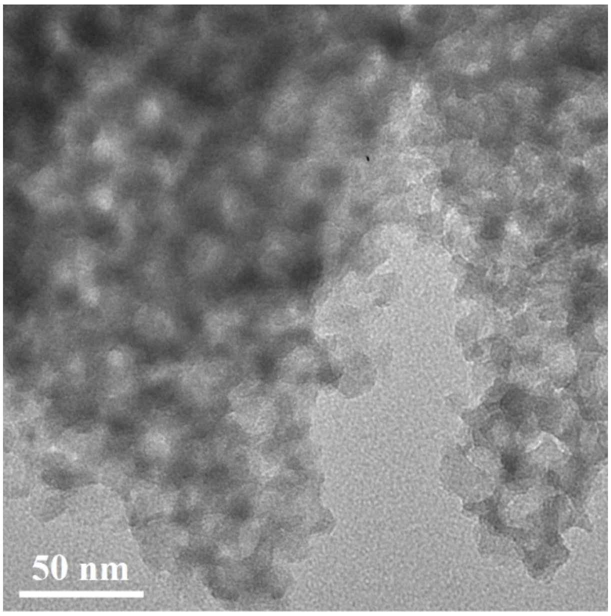

Supplementary Figure 7. TEM image of Pt_1/mpg-C_3N_4.

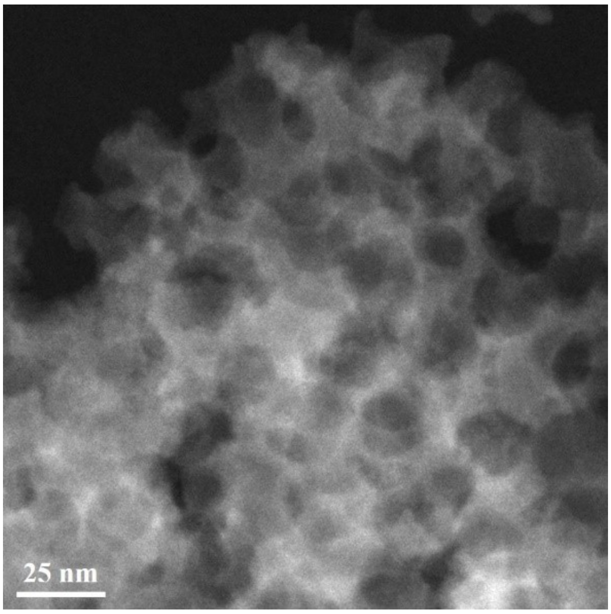

Supplementary Figure 8. HAADF-STEM image of Pt_1/mpg-C_3N_4.

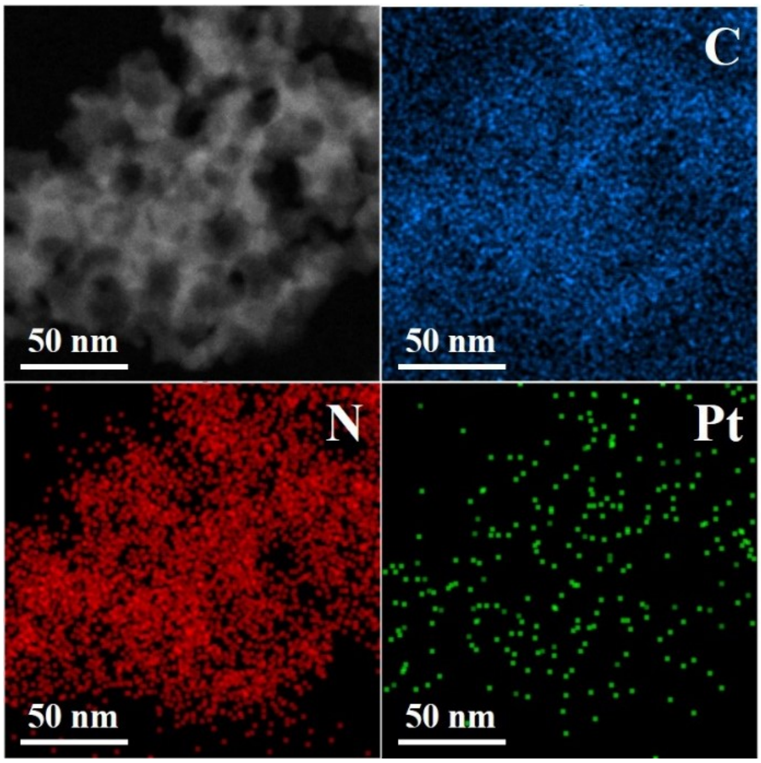

Supplementary Figure 9. Energy dispersive X-ray (EDX) elementary mapping of Pt_1/mpg-C_3N_4.

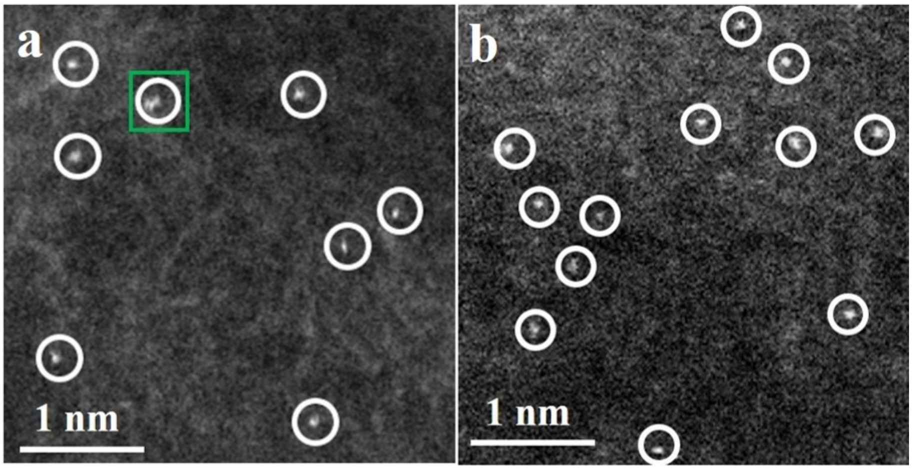

Supplementary Figure 10. (a and b) AC HAADF-STEM images of the Pt_1/mpg-C_3N_4 sample obtained in different regions. There are paried bright dots in Supplementary Figure 10a as marked within the green rectangle.

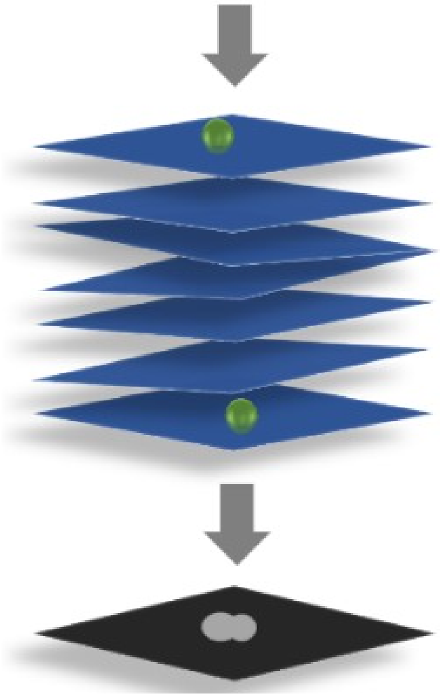

Supplementary Figure 11. The two-dimensional projection of three-dimensional sample along the incident beam direction in the AC HAADF-STEM characterization.

In Supplementary Figure 10a, there was paried bright dots (marked in the green rectangle) observed in the AC HAADF-STEM image of the $Pt_1/mpg\text{-}C_3N_4$ sample. Very probably, this feature come from two Pt single atoms, which were far away from each other in the three-dimensional space, but happened to be very close when being projected onto a certain two-dimensional plane, as shown in the Supplementary Scheme S1. This is because the AC HAADF-STEM image only represents a two-dimensional projection of a three-dimensional sample along the incident beam direction. It should be noted that the emergence of such situation is very rare. Besides, since there was no Pt–Pt path observed in the EXAFS measurements for the $Pt_1/mpg\text{-}C_3N_4$ sample, we could exclude the existence of the Pt_2 species in the $Pt_1/mpg\text{-}C_3N_4$ sample.

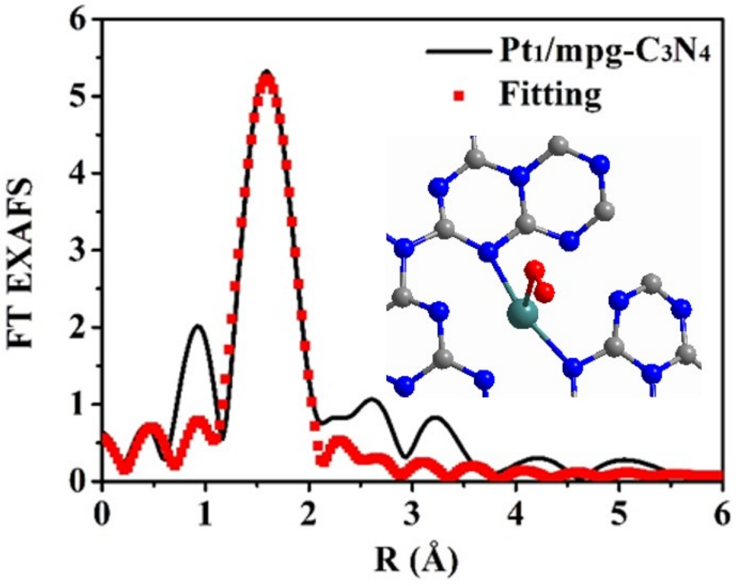

Supplementary Figure 12. FT EXAFS fitting spectrum of Pt_1/mpg-C_3N_4 at R space. The inset is a schematic model of the Pt_1/g-C_3N_4 system. The teal, gray, blue, and red spheres represent the Pt, C, N, and O atoms, respectively.

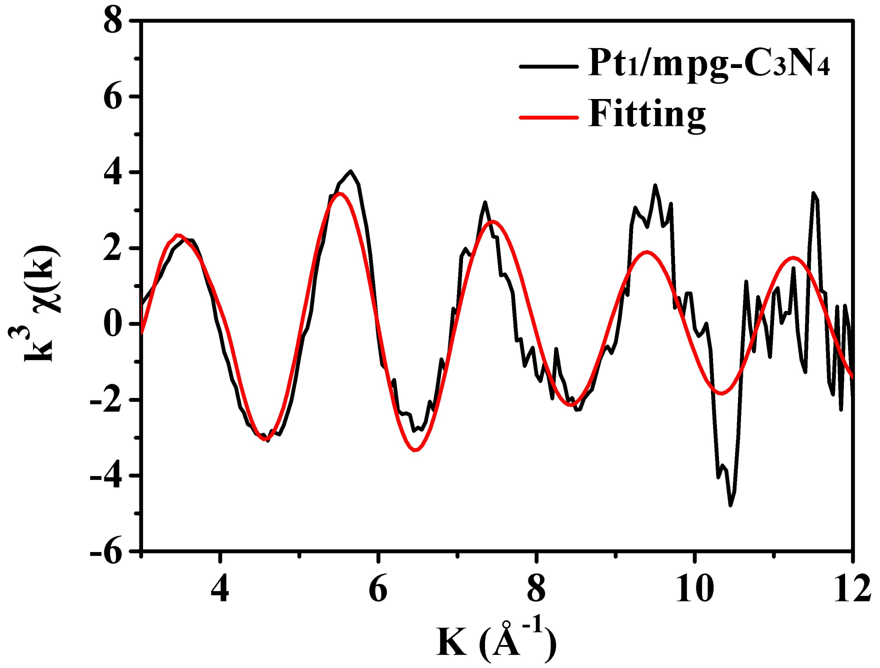

Supplementary Figure 13. FT EXAFS fitting spectrum of Pt_1/mpg-C_3N_4 at K space.

Supplementary Table 1. Pt L_3-edge EXAFS curve Fitting Parameters

sample	shell	N	R (Å)	σ^2 (Å^2)	ΔE_0 (eV)	R, %
Pt_2/mpg-C_3N_4	Pt-N(O)	2.4	2.02	5.5	6.1	0.002
Pt_1/mpg-C_3N_4	Pt-N(O)	3.1	2.04	2.8	5.4	0.001

N, coordination number; R, distance between absorber and backscatter atoms; σ^2, Debye–Waller factor to account for both thermal and structural disorders; ΔE_0, inner potential correction; R factor (%) indicates the goodness of the fit. Error bounds (accuracies) that characterize the structural parameters obtained by EXAFS spectroscopy were estimated as $N \pm 20\%$; $R \pm 1\%$; $\sigma^2 \pm 20\%$; $\Delta E_0 \pm 20\%$.

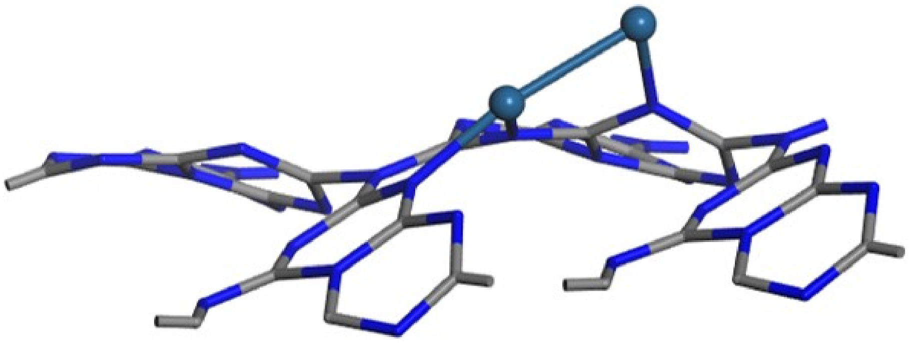

Supplementary Figure 14. Side view of Pt_2/g-C_3N_4 structure, showing the distortion of the g-C_3N_4 substrate with obvious undulations. The teal, gray, and blue spheres represent the Pt, C, and N atoms, respectively.

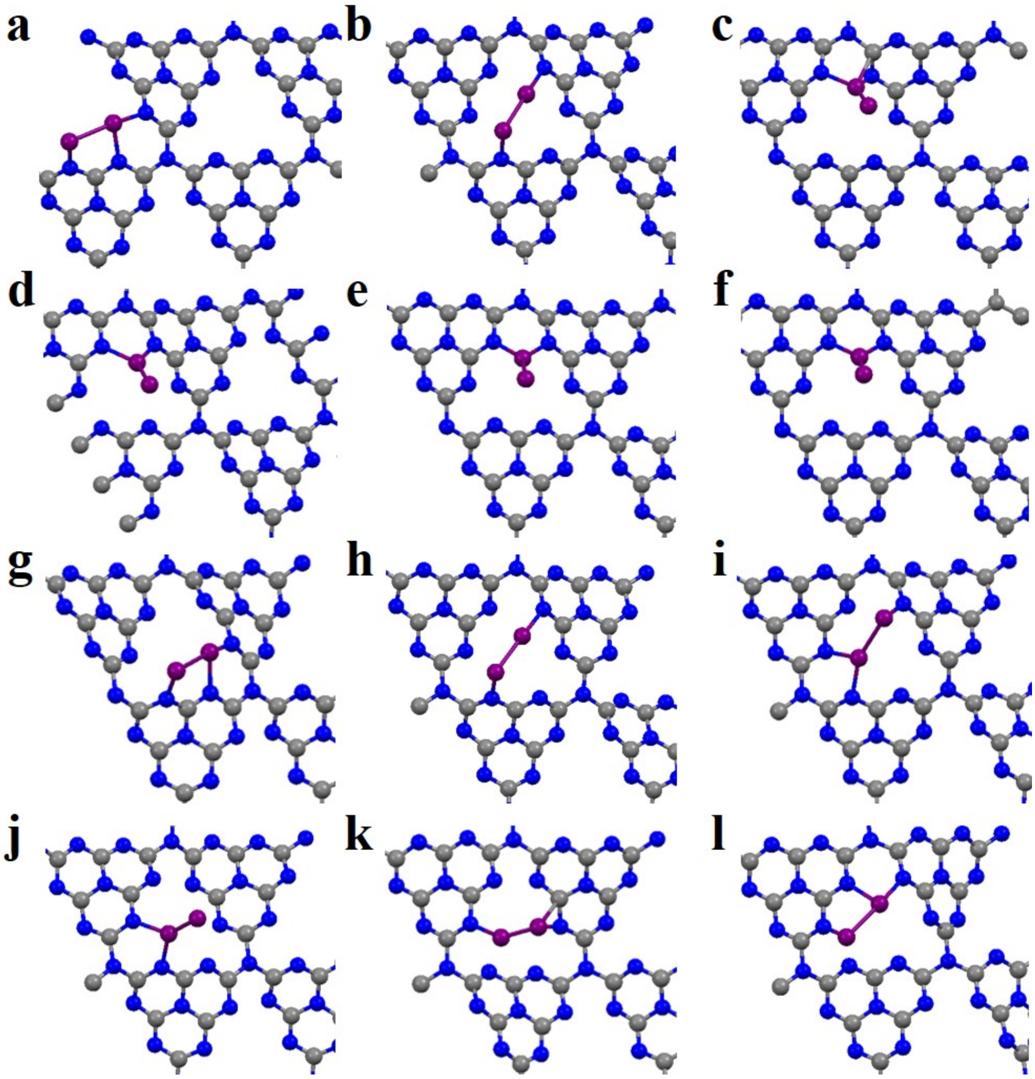

Supplementary Figure 15. Selected Pt_2 configurations (without the involvement of oxygen) on the g-C_3N_4 substrate obtained from the first-principles simulations

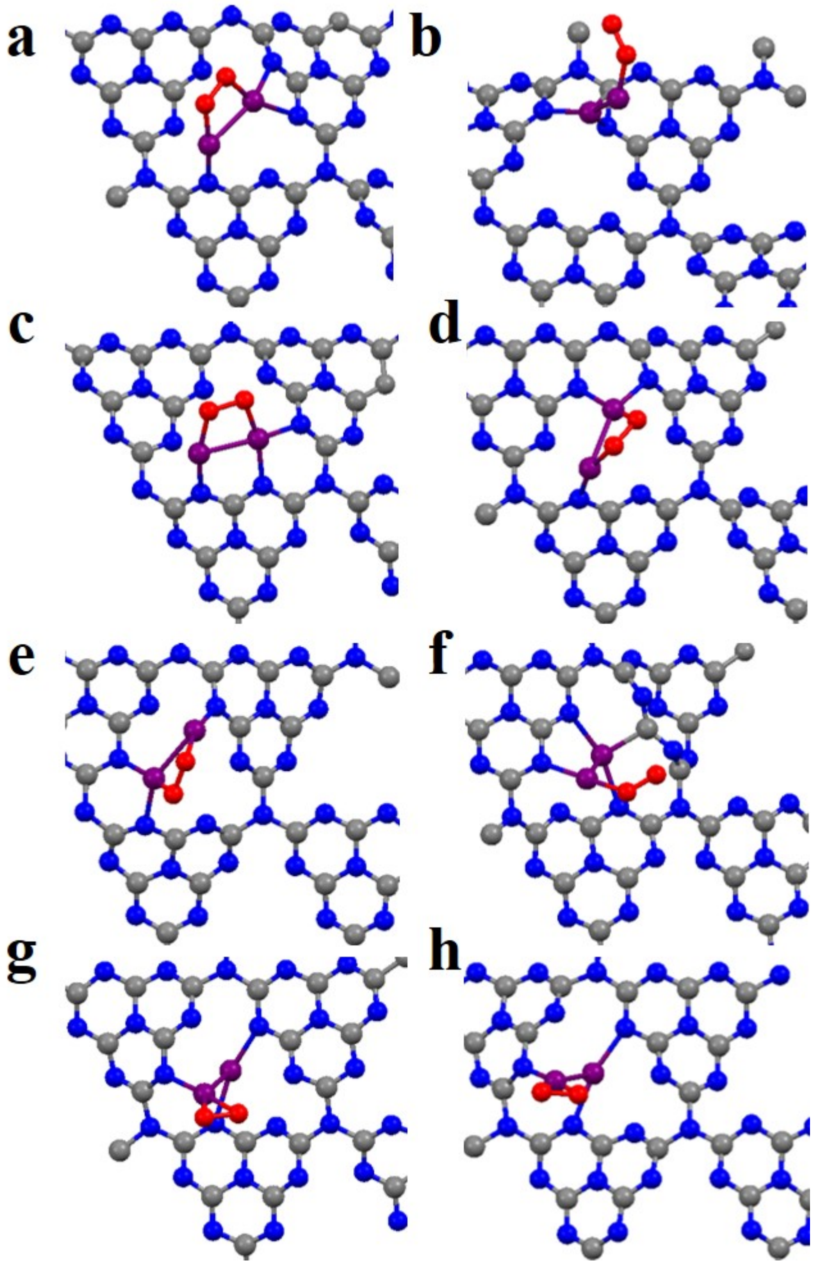

Supplementary Figure 16. Selected Pt_2-O_2 configurations (upon the associative adsorption of O_2) on the g-C_3N_4 substrate obtained from the first-principles simulations.

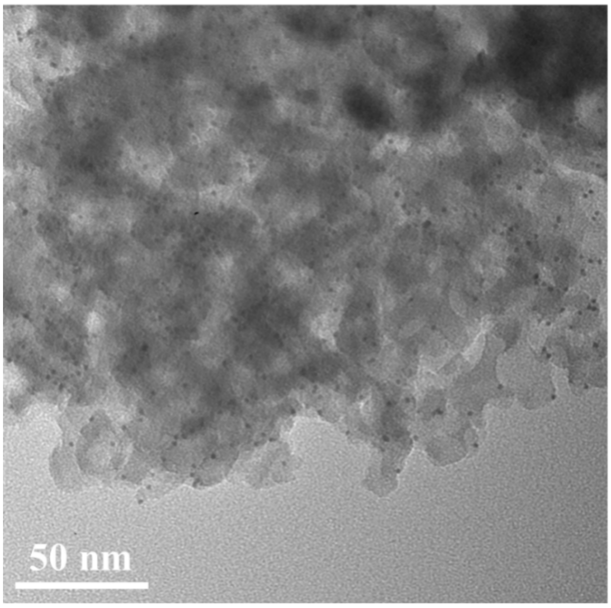

Supplementary Figure 17. TEM image of Pt NPs/mpg-C_3N_4 before the reaction.

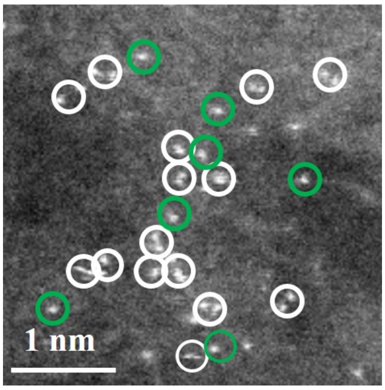

Supplementary Figure 18. AC HAADF-STEM image of Pt_2/mpg-C_3N_4 after the reaction.

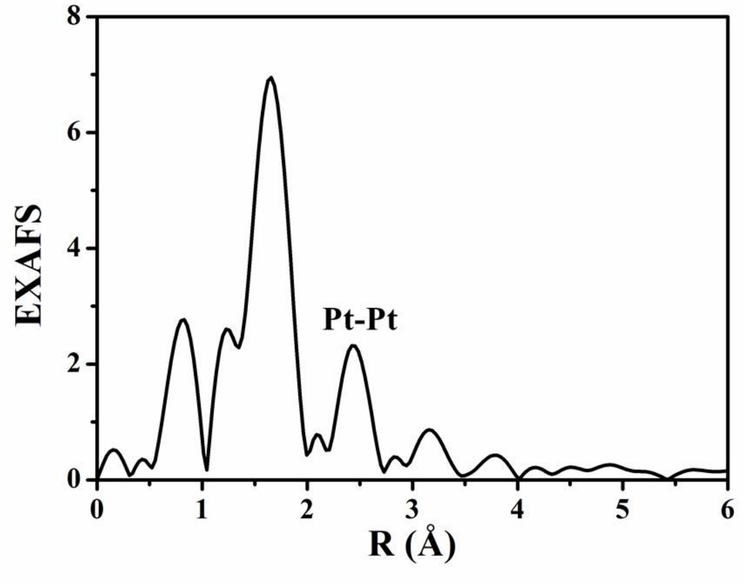

Supplementary Figure 19. EXAFS spectrum of Pt_2/mpg-C_3N_4 after the reaction.

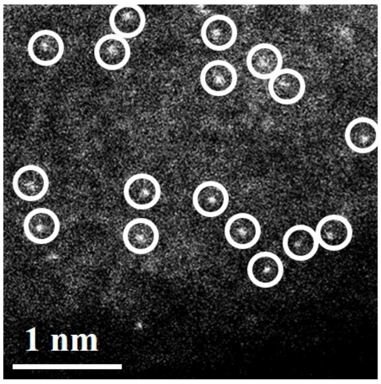

Supplementary Figure 20. AC HAADF-STEM image of the Pt_1/mpg-C_3N_4 after the reaction.

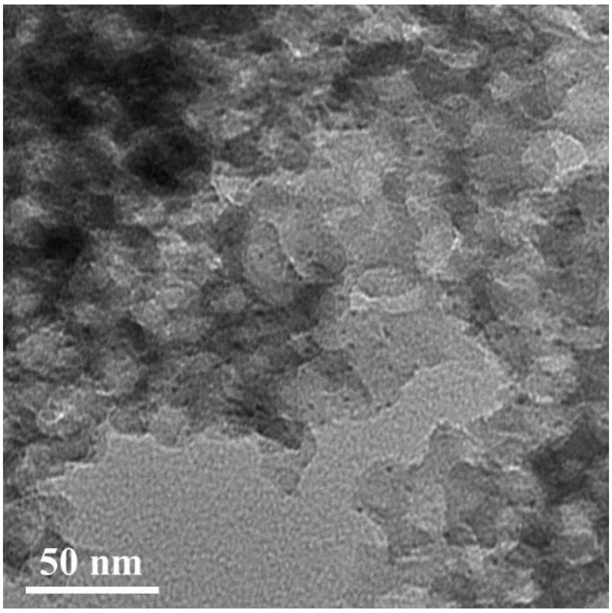

Supplementary Figure 21. TEM image of Pt NPs/mpg-C_3N_4 after the reaction.

Supplementary Table 2. Details about the hydrogenation reactions of nitrobenzene, p-nitrophenol, p-nitrotoluene, tetrachloro-nitrobenzene, and tetrabromonitrobenzene catalyzed by the $Pt_2/mpg\text{-}C_3N_4$ sample.

Catalyst	Substrate	Temperature (°C)	Time (h)	Selectivity (%)	Yield (%)
$Pt_2/mpg\text{-}C_3N_4$	nitrobenzene	100	4	100	100
$Pt_2/mpg\text{-}C_3N_4$	p-nitrophenol	100	4	100	100
$Pt_2/mpg\text{-}C_3N_4$	p-nitrotoluene	100	4	100	100
$Pt_2/mpg\text{-}C_3N_4$	tetrachloro-nitrobenzene	100	4	100	93
$Pt_2/mpg\text{-}C_3N_4$	tetrabromonitrobenzene	100	4	100	96

Standard reaction conditions: nitrocompound (1 mmol), catalyst: $Pt_2/mpg\text{-}C_3N_4$ (equal 0.000373 mmol Pt), isopropanol (10.0 mL) as solvent, T = 100 °C, t = 4 h. Determined by gas chromatography (GC) analysis with n-octanol as internal standard.

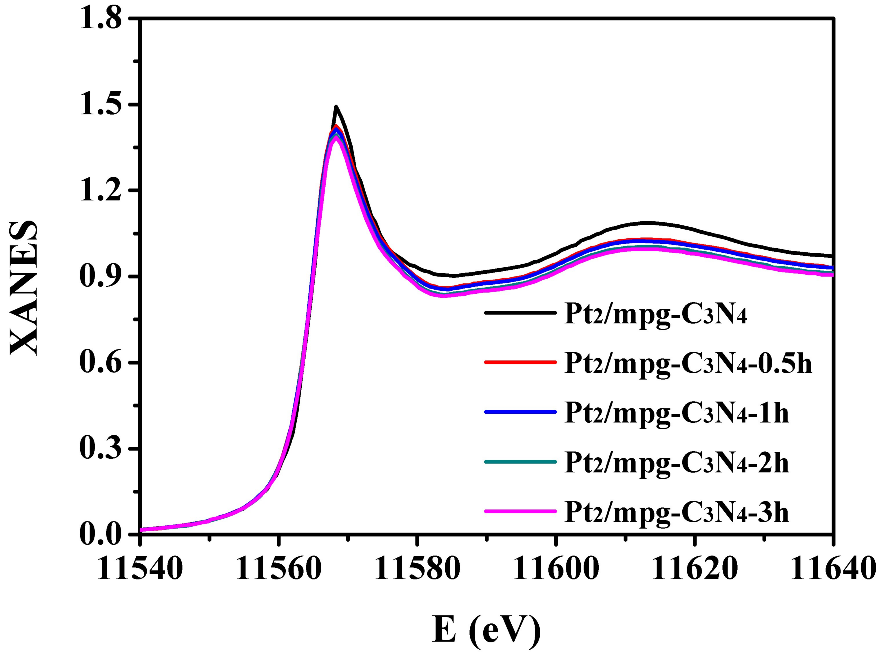

Supplementary Figure 22. Time-dependent XANES spectra of Pt_2/mpg-C_3N_4 during the hydrogenation of nitrobenzene to aniline.

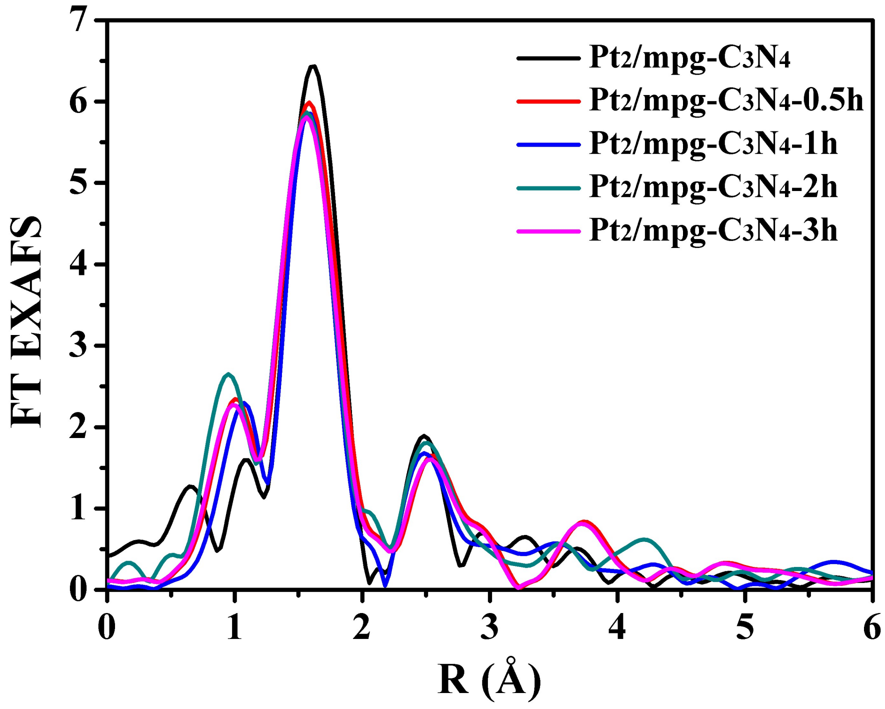

Supplementary Figure 23. Time-dependent EXAFS spectra of Pt_2/mpg-C_3N_4 during the hydrogenation of nitrobenzene to aniline.

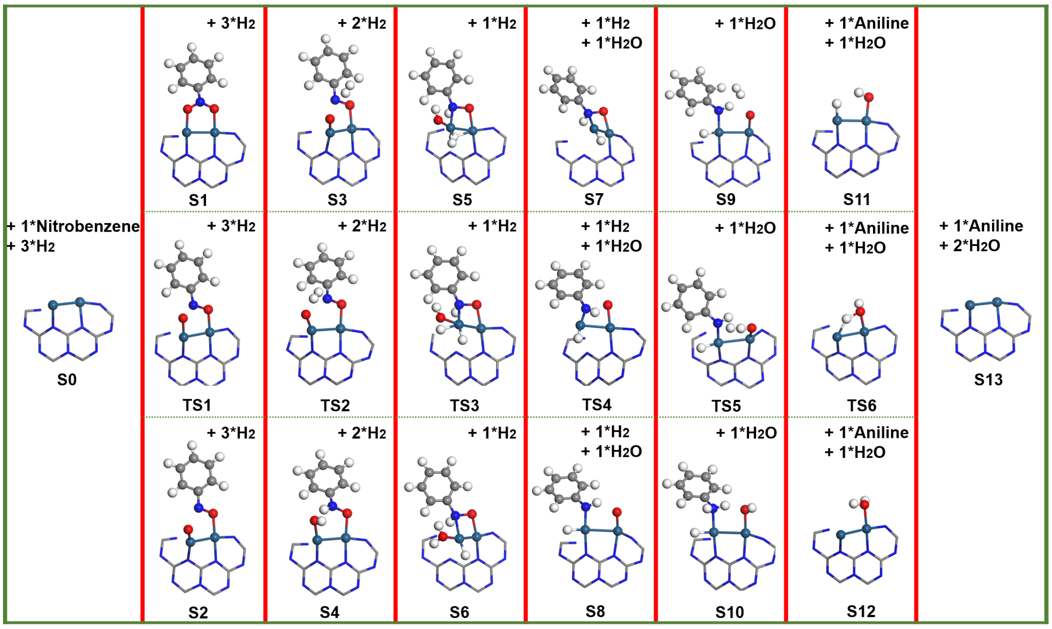

Supplementary Figure 24. Compositions and geometries of the initial state (S0), the series of the intermediate states (S1 – S12), and the series of the transition states (TS1 – TS6) in the hydrogenation reaction of nitrobenzene on the Pt_2/g-C_3N_4 catalyst (see Fig. 4 in the main text). Here, key information regarding reactant molecules which have not been adsorbed and/or product molecules which have been desorbed are also labelled. The teal, gray, blue, red, and white spheres represent the Pt, C, N, O, and H atoms, respectively.

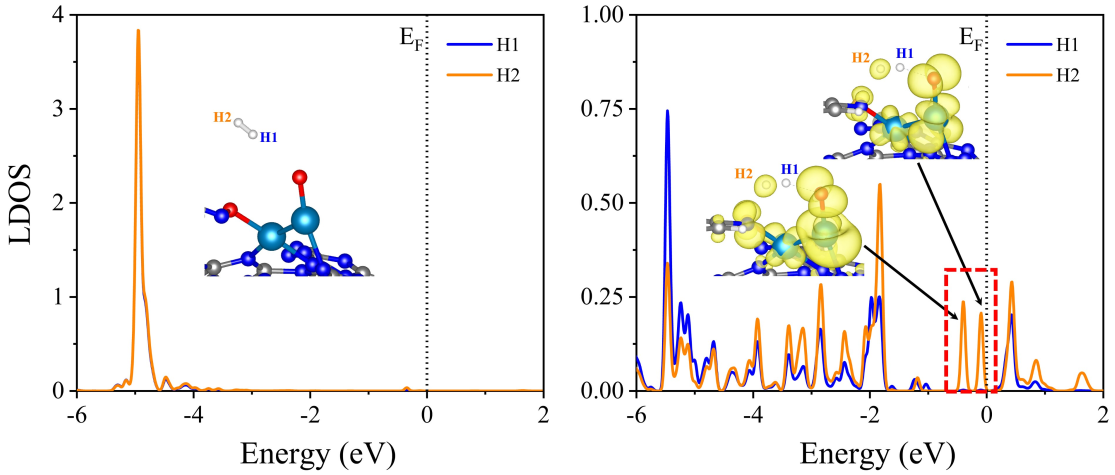

Supplementary Figure 25. Calculated electronic density of state (DOS) of the two H atoms (H1 and H2) in the S3 (left panel) and TS2 (right panel) configurations in Fig. 4, corresponding to the initial state and the transition state of the H_2 dissociation step. The teal, gray, blue, red, and white spheres represent the Pt, C, N, O, and H atoms, respectively.

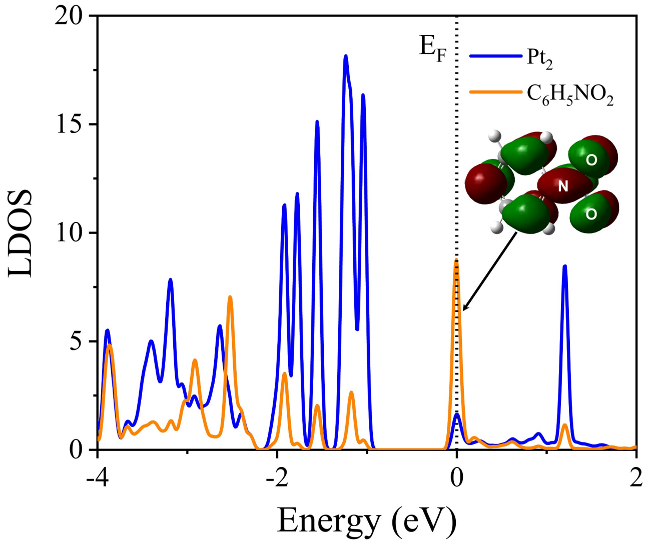

Supplementary Figure 26. Calculated electronic density of state (DOS) of $Pt_2/g-C_3N_4$ upon the adsorption of nitrobenzene. The LUMO orbital of an isolated nitrobenzene molecule is placed in the inset.

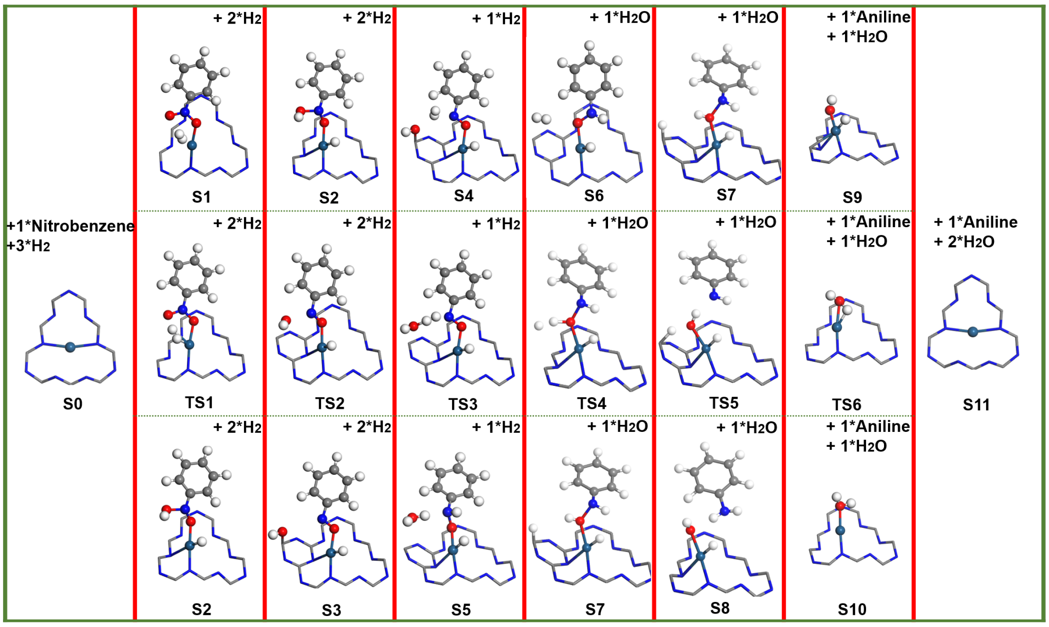

Supplementary Figure 27. Compositions and geometries of the initial state (S0), the series of the intermediate states (S1 – S10), and the series of the transition states (TS1 – TS6) in the hydrogenation reaction of nitrobenzene on the $Pt_1/g\text{-}C_3N_4$ system (see Fig. 5 in the main text). Here, key information regarding reactant molecules which have not been adsorbed and/or product molecules which have been desorbed are also labelled. The teal, gray, blue, red, and white spheres represent the Pt, C, N, O, and H atoms, respectively.

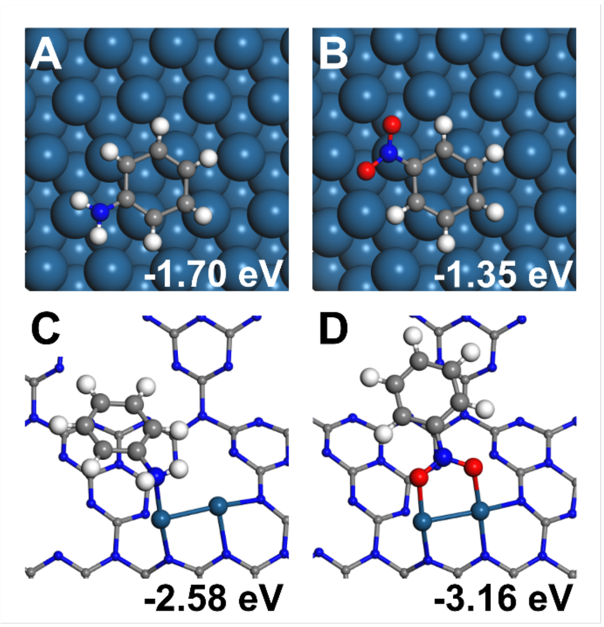

Supplementary Figure 28. Adsorption configurations of an aniline molecule (A and C) and a nitrobenzene molecule (B and D) on the Pt(111) surface (top) and the Pt_2/g-C_3N_4 catalyst (bottom). The corresponding adsorption energy values are displayed in the respective lower right corners. The teal, gray, blue, red, and white spheres represent the Pt, C, N, O, and H atoms, respectively.

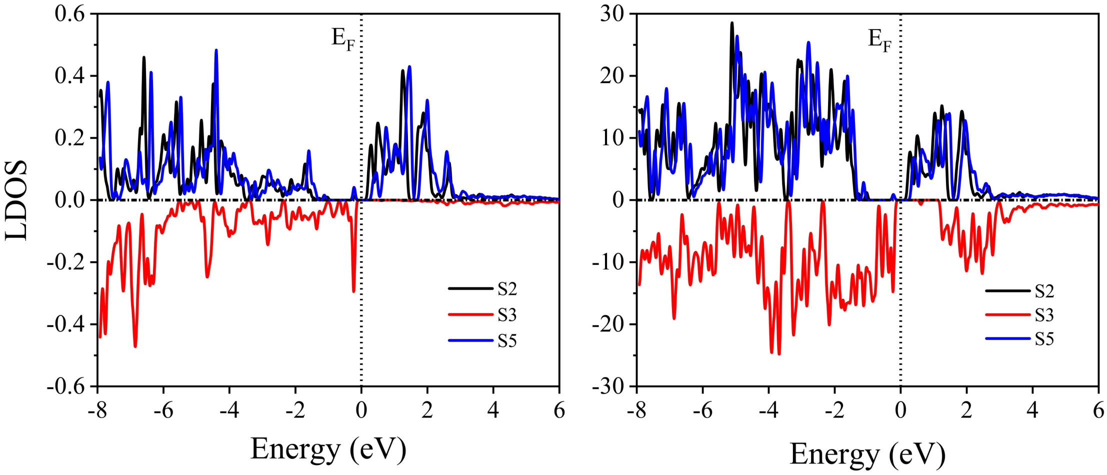

Supplementary Figure 29. Calculated electronic density of state (DOS) of the carbon atom bound to OH (left panel) and the entire g-C_3N_4 framework (right panel) for the configurations S2 (before OH connects to the C atom), S3 (with OH bound to the C atom), and S5 (after OH leaves the C atom) in Fig. 5.

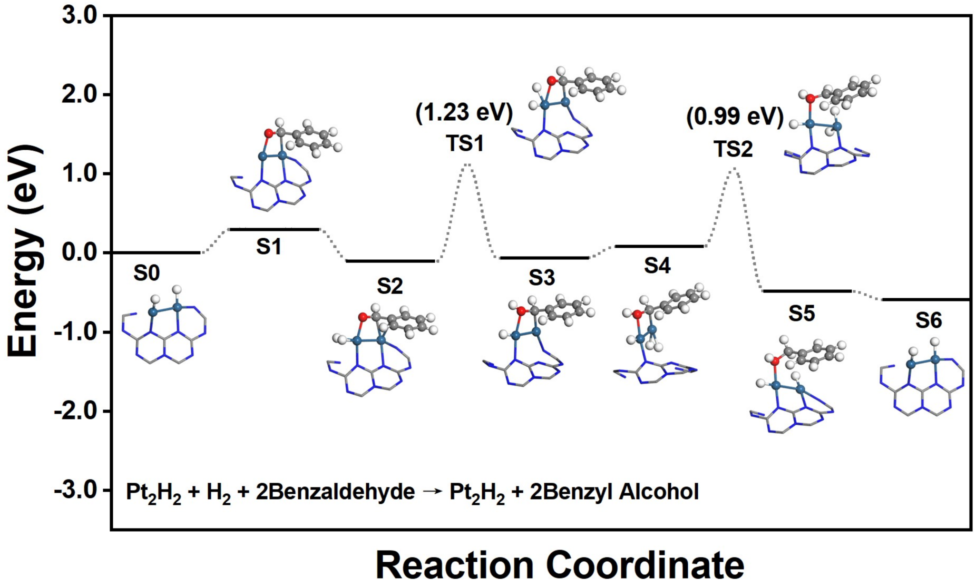

Supplementary Figure 30. Reaction pathway and computational energy profile of the hydrogenation of benzaldehyde on the Pt_2/g-C_3N_4 catalyst. The label S0 represents the initial state and the subsequent labels S1 – S6 represent a series of intermediate states. The labels TS1 and TS2 represent a series of transition states. Here, only the key structures, i.e., the Pt_2 catalytic system as well as the adsorbate bound on it, are shown. The information regarding reactant molecules which have not been adsorbed and/or product molecules which have been desorbed are labelled in the Supplementary Figure 27. The teal, gray, blue, red, and white spheres represent the Pt, C, N, O, and H atoms, respectively.

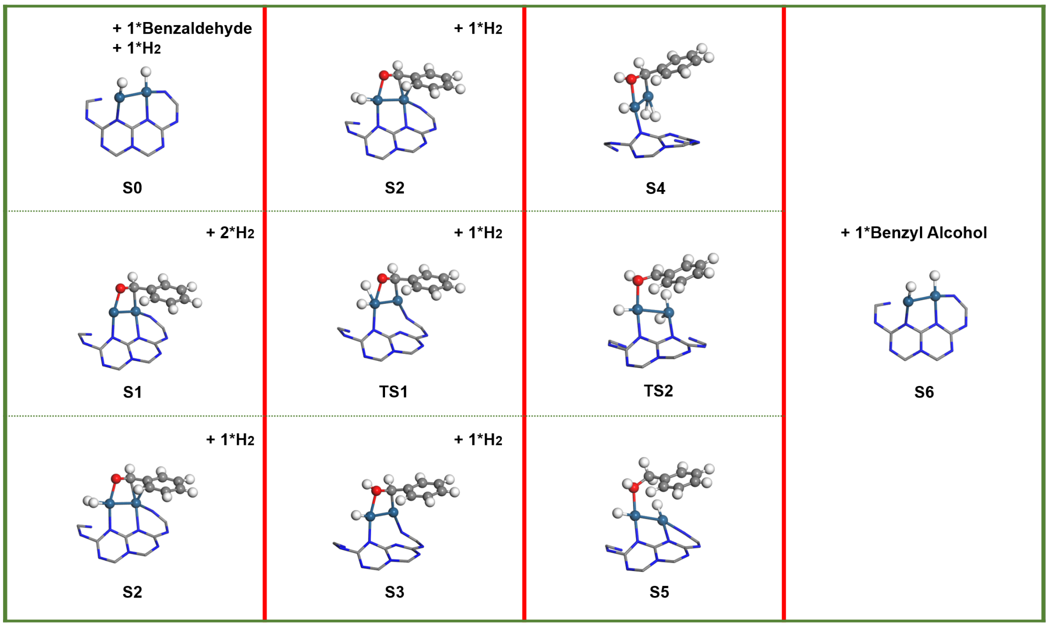

Supplementary Figure 31. Compositions and geometries of the initial state (S0), the series of the intermediate states (S1 – S6), and the two transition states (TS1 and TS2) in the benzaldehyde hydrogenation on the Pt_2/g-C_3N_4 catalyst (see Supplementary Figure 26). Here, key information regarding reactant molecules which have not been adsorbed and/or product molecules which have been desorbed are also labelled. The teal, gray, blue, red, and white spheres represent the Pt, C, N, O, and H atoms, respectively.

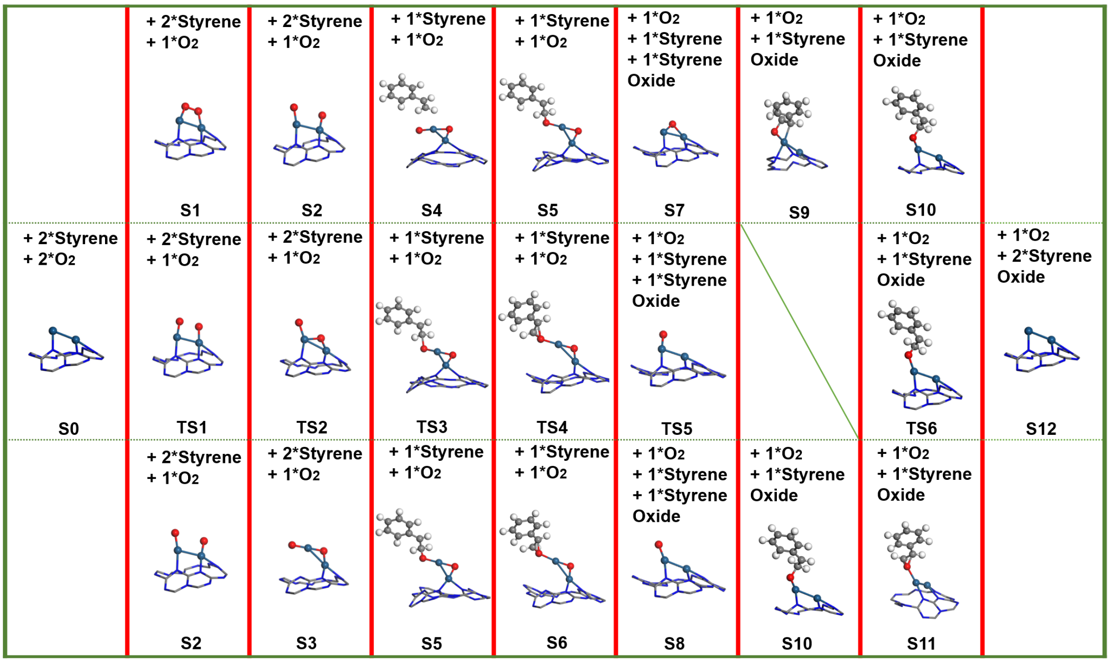

Supplementary Figure 32. Compositions and geometries of the initial state (S0), the series of the intermediate states (S1 – S11), and the series of the transition states (TS1 – TS6) in the styrene epoxidation on the $Pt_2/g\text{-}C_3N_4$ catalyst (see Fig. 7 in the main text). Here, key information regarding reactant molecules which have not been adsorbed and/or product molecules which have been desorbed are also labelled. The teal, gray, blue, red, and white spheres represent the Pt, C, N, O, and H atoms, respectively.

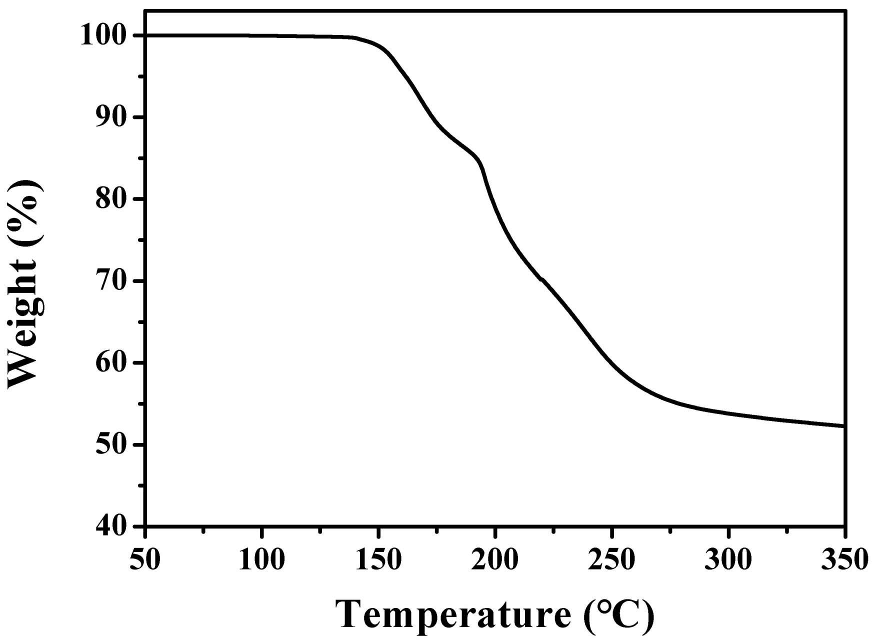

Supplementary Figure 33. TGA of Pt_2 precursor.

Supplementary Table 3. Imaginary frequencies of the transition states for the energy profile of the nitrobenzene hydrogenation on $Pt_2/g\text{-}C_3N_4$ as shown in Fig. 4.

	frequency (cm^{-1})
TS1	164.0 cm^{-1}
TS2	596.4 cm^{-1}
TS3	744.7 cm^{-1}
TS4	169.1 cm^{-1}
TS5	1102.5 cm^{-1}
TS6	947.8 cm^{-1}

Supplementary Table 4. Imaginary frequencies of the transition states for the energy profile of the nitrobenzene hydrogenation on $Pt_1/g-C_3N_4$ as shown in Fig. 5.

	frequency (cm^{-1})
TS1	1084.4 cm^{-1}
TS2	144.9 cm^{-1}
TS3	958.4 cm^{-1}
TS4	1424.6 cm^{-1}
TS5	1215.8 cm^{-1}
TS6	1098.7 cm^{-1}

Supplementary Table 5. Imaginary frequencies of the transition states for the energy profile of the styrene epoxidation on $Pt_2/g\text{-}C_3N_4$ as shown in Fig. 7.

	frequency (cm^{-1})
TS1	348.6 cm^{-1}
TS2	242.1 cm^{-1}
TS3	348.8 cm^{-1}
TS4	326.5 cm^{-1}
TS5	118.9 cm^{-1}
TS6	166.5 cm^{-1}

Supplementary Table 6. Imaginary frequencies of the transition states for the energy profile of the benzaldehyde hydrogenation on $Pt_2/g\text{-}C_3N_4$ as shown in Supplementary Fig. S30.

	frequency (cm^{-1})
TS1	1058.0 cm^{-1}
TS2	108.6 cm^{-1}
